# Phase Transition Liquid Metal Enabled Emerging Biomedical Technologies and Applications

**DOI:** 10.1002/advs.202306692

**Published:** 2023-12-25

**Authors:** Shang Gao, Yaxiong Yang, Aleksandra S. Falchevskaya, Vladimir V. Vinogradov, Bo Yuan, Jing Liu, Xuyang Sun

**Affiliations:** ^1^ School of Engineering Medicine Beihang University Beijing 100191 China; ^2^ Key Laboratory for Biomechanics and Mechanobiology of Ministry of Education School of Biological Science and Medical Engineering Advanced Innovation Center for Biomedical Engineering Beihang University Beijing 100083 China; ^3^ International Institute “Solution Chemistry of Advanced Materials and Technologies” (SCAMT) ITMO University Saint Petersburg 191002 Russia; ^4^ School of Mechanical Engineering and Automation Beihang University Beijing 100191 China; ^5^ Department of Biomedical Engineering, School of Medicine Tsinghua University Beijing 100084 China

**Keywords:** biomedical application, gallium‐based liquid metal, liquid metal, phase transition

## Abstract

Phase change materials that can absorb or release large amounts of heat during phase transition, play a critical role in many important processes, including heat dissipation, thermal energy storage, and solar energy utilization. In general, phase change materials are usually encapsulated in passive modules to provide assurance for energy management. The shape and mechanical changes of these materials are greatly ignored. An emerging class of phase change materials, liquid metals (LMs) have attracted significant interest beyond thermal management, including in transformable robots, flexible electronics, soft actuators, and biomedicine. Interestingly, the melting point of LM is highly tunable around body temperature, allowing it to experience considerable stiffness change when interacting with human organisms during solid–liquid change, which brings about novel phenomena, applied technologies, and therapeutic methods, such as mechanical destruction of tumors, neural electrode implantation technique, and embolization therapy. This review focuses on the technology, regulation, and application of the phase change process along with diverse changes of LM to facilitate emerging biomedical applications based on the influences of mechanical stiffness change and versatile regulation strategies. Typical applications will also be categorized and summarized. Lastly, the advantages and challenges of using the unique and reversible process for biomedicine will be discussed.

## Graphical abstract

Phase transition of liquid metals that occurs around body temperature, brings amounts of interesting phenomena and holds huge prospects in biomedical area. This review is dedicated to the unique bidirectional conversion of liquid metals, highlighting these unique changes during phase transition, classifying the stimulaiton methods, elaborating these emerging biomedical applications enabled by phase transition, and outlining future scenarios in biomedicine.

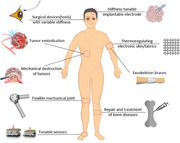

## 1 Introduction

The development of smart materials and emerging technologies has contributed to significant advances in medicine, diagnostics, and therapeutics.^[^ ^1^ ^]^ Stimuli‐responsive materials capable of responding to environmental changes have aroused great interest across several areas, including intelligent control, flexible actuators, and controlled drug release.^[^ ^2^ ^]^

As new generation of smart materials, liquid metals (LMs;, i.e., gallium (Ga), indium (In), bismuth (Bi), and their alloys) possess many impressive properties, such as high biocompatibility, excellent thermal and electrical conductivity, self‐healing ability, water‐like fluidity, low viscosity, and low vapor pressure,^[^ ^3^ ^]^ leading to many unique phenomena and featured applications in soft robots,^[^ ^4^ ^]^ electronic printing,^[^ ^5^ ^]^ thermal management,^[^ ^6^ ^]^ and biomedicine.^[^ ^7^ ^]^

Due to their favorable biosafety and low elastic modulus in liquid phase under a normal atmosphere, LMs exhibit excellent flexibility and conformability, which makes them suitable for use in organisms and for interaction with biological tissues.^[^ ^8^ ^]^ In the biomedical field, LMs offer a potential solution to a myriad of complex biomedical problems, such as the treatment of tumors, nerve connection, angiography, flexible sensors,^[^ ^9^ ^]^ electronic skin,^[^ ^10^ ^]^ health monitoring,^[^ ^11^ ^]^ and antibacterial applications.^[^ ^12^ ^]^ Considerable effort has been devoted to the development of LM‐based soft conductive materials and composites with ultra‐low elastic modulus, highly stretchable devices with reduced mechanical mismatch between the curvilinear skin, and the monitoring healthcare sensors.^[^ ^13^ ^]^ In addition, LMs can be easily dispersed into large amounts of nanoparticles in a top‐down method to serve as a novel theranostic platform for biomedical imaging as well as disease treatments, including drug delivery, laser ablation, cryoablation, endosome escape, and even pathogen treatment.^[^ ^14^ ^]^ Ga can disrupt iron‐dependent metabolic pathways in bacteria, inhibiting their growth. When exposed to magnetic fields, LM nanoparticles can transform, develop sharp edges, and destroy the protective bacterial biofilm.^[^ ^15^ ^]^

Metals conventionally used in biomedicine (e.g., Mg, iron (Fe), and Ti) tend to have high melting points, which makes it difficult to explore their phase change behaviors, let alone take advantage of their phase transitions and their potential biomedical applications.^[^ ^16^ ^]^ LMs provide a feasible and safe platform to study metallic phase changes and their accompanying biomedical applications, and represent a large class of functional materials imbued with tunable melting points, which is accomplished by manipulating their elemental compositions. To date, a substantial number of materials with melting points around human body temperature have been developed.^[^ ^17^ ^]^ In fact, it is worth noting that one of the main advantages of LMs is their low melting points, allowing low and available energy to trigger the phase transitions, a property that is greatly ignored but has enormous potential, especially in biomedical fields.

**Figure 1:**
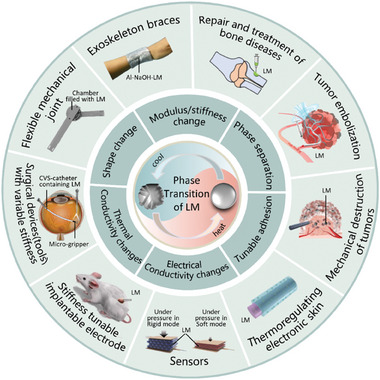
Liquid metal (LM) phase transition‐induced changes in as well as the emerging biomedical technologies and applications endowed by the phase transition process. Under cooling, such as nature cooling, ice cooling or liquid nitrogen cooling, LM can achieve phase transition from liquid to solid state. On the other hand, under mild heating, such as body temperature heating, water heating, or Joule heating, it can induce LM to change state from solid to liquid state. The phase transition‐induced changes include shape change, modulus/stiffness change, phase separation, tunable adhesion, and changes in thermal and electrical conductivity. The emerging biomedical technologies and applications include flexible mechanical joints, exoskeleton wearables, bone repair and treatment of bone diseases, tumor embolization, mechanical destruction of tumors, thermoregulating electronic skin, sensors, stiffness tunable implantable electrodes, and surgical devices with variable stiffness. Reproduced with permission.^[^ ^18a^ ^]^ Copyright 2014, American Society of Mechanical Engineers. Reproduced with permission.^[^ ^18b^ ^]^ Copyright 2020, Elsevier. Reproduced with permission.^[^ ^18c^ ^]^ Copyright 2021, Wiley‐VCH. Reproduced with permission.^[^ ^18d^ ^]^ Copyright 2022, Wiley‐VCH. Reproduced with permission.^[^ ^18e^ ^]^ Copyright 2021, Elsevier. Reproduced with permission.^[^ ^18f^ ^]^ Copyright 2022, Wiley‐VCH. Reproduced with permission.^[^ ^18g^ ^]^ Copyright 2022, Elsevier. Reproduced with permission.^[^ ^18h^ ^]^ Copyright 2020, Wiley‐VCH. Reproduced with permission.^[^ ^18i^ ^]^ Copyright 2021, Wiley‐VCH.

This review focuses on the unique phase transition property of LMs as well as the emerging biomedical technologies and applications endowed by the phase transition process (**Figure** **1**). The melting of LM can be easily achieved in a mild environment. When the ambient temperature is above the melting point, the metal changes from solid to liquid state. When the temperature is decreased, the metal solidifies transitioning from the liquid to the solid state. The changes of LMs triggered by phase transition were illustrated and the stimulation methods were classified. The medical applications based on phase transitional LMs were summarized. Finally, current perspectives and future outlooks on this field are also discussed.

## 2 Fundamental Aspects of LMs

LMs are distinguished by their high electrical and thermal conductivity, low viscosity, and high surface tension.^[^ ^19^ ^]^ They also have unique properties, including the capability to wet most materials, which makes them useful in various industrial applications. LM behavior is influenced by several factors, including temperature, pressure, and composition. LM composition can also affect their properties, with some alloys exhibiting improved mechanical strength or corrosion resistance.

LMs, which consist of post‐transition metals (Ga, In, tin (Sn), and Bi), zinc (Zn)‐group metals (Zn, cadmium (Cd), and mercury (Hg)), and alkali metals (lithium (Li), sodium (Na), potassium (K), rubidium (Rb), cesium (Cs), francium (Fr)) are metals with melting temperatures under 300 °C.^[^ ^20^ ^]^ Generally, the melting point of metals is affected by many factors, namely the number of electrons involved in the bond, degree of valence electron delocalization, and crystal structure in the solid state. For instance, Zn‐group metals have relatively low melting points due to their electronic configurations, with only two valence electrons involved in bonding as well as complete *s* and *d* subshells (pseudo “noble gas” electron configuration).^[^ ^20a^ ^]^ Like Zn group metals, the low melting points of alkali metals can also be attributed to their electronic configuration; however, their large atomic radii result in weak metallic bonding within the metal. Post‐transition metals have low melting points due to their partially filled *p* subshells, which enables them to form covalent or metallic bonds, resulting in the metal remaining in a liquid state over a wide temperature range. Of these metals, Ga and Ga‐based alloys are especially interesting because of their vast range of potential applications in the energy sector, the manufacture of flexible devices, and medicine.^[^ ^21^ ^]^

**Figure 2:**
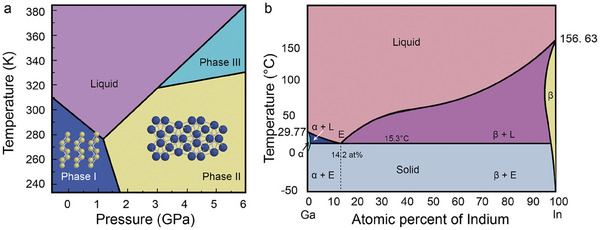
Phase diagram of LMs. a) Phase diagram of Ga depicting α‐Ga and Ga II (covalent bonds between Ga in Ga_2_ are drawn in red). Adapted from.^[^ ^26^ ^]^ Copyright 1991, American Physical Society. b) Phase diagram of the EGaIn alloy, the α and β regions correspond to the regions of existence of homogeneous solid solutions In in Ga (α) and Ga in In (β), respectively, the composition of which depends on temperature, and E for eutectics. Adapted from.^[^ ^29^, ^30^ ^]^ Copyright 1998, American Physical Society; Copyright 2022, Springer Nature.

Thanks to its unique crystal structure, Ga exhibits distinct physical and chemical properties. At 20 °C, solid Ga has a density of 5.904 g cm^−3^ while liquid Ga has a density of 6.095 g cm^−3^.^[^ ^22^ ^]^ This indicates that Ga, unlike most other substances, expands when it changes from a liquid to a solid state. This property is quite rare and occurs in only very few substances (e.g., Si, Bi, Ge, and water). Like all post‐transition metals, Ga can exist as a liquid over a very wide temperature range (almost 2200 K), with a melting (crystallization) point of 302.93 K and a boiling point of 2477 K. The low melting point of Ga makes it easy to transition the metal between its solid and liquid states and observe abrupt property changes near the equilibrium melting temperature.^[^ ^16^ ^]^ So far, at least eight different types of solid Ga structures have been identified. Only one of these polymorphic modifications, ɑ‐Ga, is thermodynamically stable at ambient pressure while the others (i.e., β‐Ga, γ‐Ga, δ‐Ga, and ε‐Ga) are unstable.^[^ ^23^ ^]^ α‐Ga has a primitive orthorhombic crystal lattice with eight atoms (**Figure** **2**a). Each atom is coordinated to seven neighbors with the closest neighboring atom being 2.44 Å away and the remaining six atoms being located 2.71 to 2.79 Å away.^[^ ^24^ ^]^ Studies have shown that ɑ‐Ga is a metal molecular crystal with a strong Ga_2_ covalent bond and weaker intermolecular forces.^[^ ^25^ ^]^ Diatomic dumbbell‐like quasi molecules (Ga_2_) can be seen in the layered rhombic lattice of Ga, contributing to structural anisotropy, which can be disrupted at lower temperatures. Ga has been observed to remain in a liquid state at both the micrometer and submicrometer scale, even when undercooled to temperatures as low as 150 K. However, undercooling results in the formation of β‐Ga, which has a monoclinic structure packed in parallel layers,^[^ ^26^ ^]^ instead of the stable α‐Ga phase.^[^ ^27^ ^]^ With an increase in pressure, two more polymorphic Ga structures can be observed: Ga II and Ga III, which have cubic and tetragonal lattices, respectively.^[^ ^26^ ^]^ Furthermore, during the transformation of solid α‐Ga to liquid Ga, Ga‐IV (fcc) and Ga‐V (hR6) phases may emerge, depending on the specific pressure and temperature conditions.^[^ ^28^ ^]^

An effective way of tuning the properties of LMs is through alloying. Ga forms alloys with most metals and metalloids.^[^ ^29^ ^]^ While different Ga‐metal/metalloid combinations produce different types of alloys, eutectic Ga alloys, namely Ga‐In and Ga‐In‐Sn, are the most widely studied. Ga‐In and Ga‐In‐Sn have eutectic temperatures of 15.3 and 13.2 °C, respectively.^[^ ^20a^ ^]^ The Ga‐In phase diagram falls under the category of diagrams that exhibit complete solubility in the liquid phase but restricted solubility in the solid phase.^[^ ^30^ ^]^ The amount of In that can dissolve in Ga when solid is only ≈0.3 mol.%, forming a Ga‐based α solid solution. Conversely, when In solidifies, the amount of Ga that can dissolve in it is roughly 2.2 mol.%, resulting in an In‐based β solid solution at the eutectic temperature. The solidus line on the In side of the diagram shows a clear retrograde behavior, and at ≈325 K, the amount of Ga that can dissolve in In increases to 3.1 mol.%.^[^ ^29a^ ^]^ The addition of In and Sn to Ga results in a more disordered structure due to the expansion of the cell volume and an increase in interatomic distance caused by their larger atomic volume, resulting in a decrease in the melting point of the alloy.^[^ ^31^ ^]^

In addition, Ga possesses high surface tension that is almost ten times that of water.^[^ ^32^ ^]^ The excellent thermal and electrical conductivity, which is inherent from metals, renders them widely used in thermal management, flexible electronics, and E‐tattoos. Ga can form a layer thick of surface oxide only several nanometers under ambient conditions, which is often utilized in the development of large‐scaled 2D materials and optoelectronic devices.^[^ ^33^ ^]^

Chemical reactions between LMs and other transition metals, such as Aluminum (Al), Copper (Cu), and silver (Ag) play an important role in the development of composite materials, soft motors, and selective printing.^[^ ^34^ ^]^ Particular attention should be paid to the corrosion problems and a layer of encapsulation can protect LMs from leakage and other chemical contamination. As novel biomedical materials, Ga‐based alloys are generally considered to have low toxicity, however, there are still concerns regarding long‐term exposure.^[^ ^35^ ^]^

## 3 Phase Transition‐Induced Changes in LMs

Distinguished from traditional metal materials, LM phase transition is easy to achieve through mild means, and can induce many unusual changes in LM properties, such as changes in shape, modulus, phase separation, adhesion, and electrical and thermal conductivity. These properties would be discussed in detail in this section.

### 3.1 Shape Change

In the liquid state, shape transformation allows LMs to exhibit versatile morphologies, which has attracted considerable interest by research focused on soft actuators, amorphous electrodes, and flexible sensors.^[^ ^36^ ^]^ In the solid state, the shapes of LMs are usually fixed, which allows them to perform specific tasks, such as mechanical support, safety protection, and stable interaction. Phase transformations whether from liquid to solid or solid to liquid affect the shape of LMs. This process depends on the inherent properties of a particular LM, such as the interatomic distance and lattice size in different phase states, as well as on other influential external factors, such as the environment or exposure to electric, thermal, or magnetic energy. Once the shape transformation is tunable and controllable, it nourishes rich scientific discoveries and applications.

**Figure 3:**
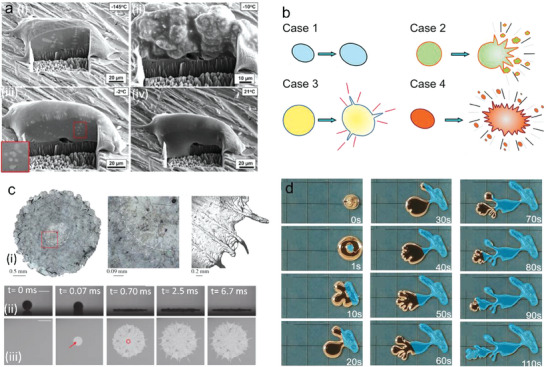
Phase transition induced shape change. a) In situ SEM imaging of fast frozen (i) and remelted (ii–iv) EGaIn droplets. Reproduced with permission.^[^ ^37^ ^]^ Copyright 2022 Elsevier. b) Four deformable behaviors of LM droplet, including uniform‐sized expansion (case 1), deformation from a single point (case 2), deformation from multiple points (case 3) and comprehensive deformation (case 4). Reproduced with permission.^[^ ^39^ ^]^ Copyright 2020, American Chemical Sociaty. c) Microscopic images of the solidified pattern of a Sn droplet after impacting on a sapphire substrate (i), side view image (ii), and bottom view image (iii) of a Sn droplet impact on a sapphire substrate at 150 °C. the scale bar is 2 mm. Reproduced with permission.^[^ ^40^ ^]^ Copyright 2019, Cambridge University Press. d) Images of deformation and crystallization of supercooling liquid Ga under 5 V voltage in 0.5 mol L^−1^ NaOH solution at 22 °C. Blue areas indicate frozen/solidified areas. Reproduced with permission.^[^ ^41^ ^]^ Copyright 2017, John Wiley and Sons.

Kim et al.^[^ ^37^ ^]^ explored several factors that influenced the microstructure and shape transformation of EgaIn microdroplets (i.e., cooling rate, droplet size, and Ga crystallization), and discovered anomalous shape transformation of LM during remelting. The authors fast‐cooled EgaIn microdroplets (1 mm or less in size) by immersing them in liquid nitrogen. Scanning electron microscopy (SEM) showed that a dendritic In‐rich phase formed in a dark Ga‐rich matrix phase. Upon remelting, the Ga‐rich phase expanded, which is in complete contrast to the behavior of slowly frozen EgaIn droplets (**Figure** **3**a). The observed volume expansion during heat cycling was attributed to immiscibility between the Ga and In phases and the formation of a metastable γ‐Ga phase. When quenched at a fast cooling rate, supercooling was high, leading to the formation of a metastable Ga phase. Then the entire system was transformed to a hypereutectic state due to the existence of the γ‐Ga phase. This was different from the situation of slow cooling in that α‐Ga played the dominant role and the melting temperature was higher. The mixture of metastable Ga phases contributed to the size‐effect of melting points and explains the shape expansion behavior. In addition, in Bi–Ga alloy systems, highly ordered nano‐patterns would preferentially appear on the alloy surface during solidification, and various transitions, such as hybridization and crystal defect‐like patterns, as well as layered and rod‐like structures could be observed.^[^ ^38^ ^]^

Under the stimulation of low temperature, the two‐fluid system composed of a LM microdroplet and an aqueous solution undergoes a rapid and violent transformation, in which the LM droplet can transform from ellipsoidal to an amorphous form.

Sun et al.^[^ ^39^ ^]^ discovered the unique transformation of LM droplets under freezing and investigated the potential influential conditions (Figure 3b), including cooling rate and solution composition around the droplets. The densification of ice crystals could play a significant role in the regulation of LM shape transformation. The results showed that denser ice crystals form when the cooling rate decreases or by the addition of dimethyl sulfoxide to the surrounding solution, limiting the deformation range of the LM.

Under isothermal conditions, a LM droplet colliding with a substrate shows splashing and rich dynamics (Figure 3c).^[^ ^40^ ^]^ When temperature of the substrate is lower than the melting point of the liquid droplet, solidification occurs upon impact, resulting in the droplet spreading, unstable splashing, ligament formation, and the resulting micropatterns. When a liquid Sn droplet impacted the sapphire substrate and spread out on the surface of the cold substrate, unique radial ligament patterns were formed during droplet solidification from the center of the droplets. Varying substrate temperatures triggered different impact processes and formed stripe patterns. Microscopic images showed a central black dot surrounded by the defect‐free region, which is formed from an air bubble trapped in the solidified central region due to first contact with the substrate. Outside the defect‐free region, a circular ring pattern could be observed, which was caused by the air trapped by the pinning effect during solidification at the contact line. New contact could be formed if the LM continued to spread but solidification prevented the flow. In the outermost layer, microstructures, such as circular ridges, radial stripes and ligaments were formed, which was caused by the rapid outward flow of the fluids over previously solidified patterns. The resulting patterns involve complex dynamics of fluids and phase changes of liquids.

Supercooling presents an unstable state and can induce fast crystallization via various methods. Regulating on the supercooling of LMs could rapidly trigger the crystallization process of liquid Ga at room temperature, in which the shapes of the solidified LM are tunable with an electrical field. Recently, researchers proposed reversible and irreversible deformation of supercooled liquid Ga in alkaline or acidic electrolytes using non‐wetting or liquid Ga‐wetting electrodes, respectively (Figure 3d).^[^ ^41^ ^]^ For the non‐wetting condition, the transformation of LM is reversible, however, if Ga‐coated electrode is used to induce the transformation, shape transformation and the phase change is irreversible. Under this situation, the various shape conformation of solidified patterns can be easily achieved.

### 3.2 Modulus/Stiffness Change

With low melting points, LMs can easily realize phase transition, accompanied by stiffness change around room temperature.^[^ ^42^ ^]^ To date, LMs, some LMs, namely Ga, Bi and their alloys have become an emerging class of materials with tunable stiffness and have shown immense potential in a number of areas, such as soft robots, flexible human‐machine interaction, exoskeleton support, and bionic systems.

Stiffness tunable materials are an effective solution to overcome the tradeoff between the demands of flexibility and certain loading or supportive capabilities. Compared to commonly used stiffness‐tunable materials, such as shape memory alloys, shape memory polymers (SMPs) and thermoplastic polymers, LMs are the most outstanding with super‐large stiffness variation from nearly 0 to ≈10^9^ Pa, which covers almost the entire mechanical ranges of living organisms. The unique and easily tunable properties allow them to mimic complex physiological environments of mechanical change or provide safety protection and capability enhancement of humans. In addition, with melting point near room temperature as well as excellent thermal conductivity and electrical conductivity, the power required to trigger the phase transition is extremely low.

Recent studies have taken advantage of the stiffness tunable property of LMs and developed various hybrid composites, and applied strategies for functional extraction of special uses. During stiffness change, LMs transition from solid to liquid; therefore, encapsulation is necessary to prevent leakage. Compared to LM, the common thermally responsive stiffness‐change polymers, such as epoxy undergo glass transition to decrease their mechanical strength in order for them to be easily bent or stretched, without damaging their inner polymer chains. Although epoxy can be subjected to a certain force and become flexible, the stiffness variation ratio is relatively low compared to LMs.

**Figure 4:**
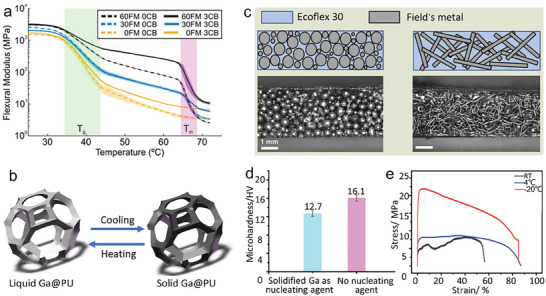
Stiffness change derived from LM phase transition. a) Flexural modulus of composites from different volume percentages of FM and carbon black. Reproduced with permission.^[^ ^43^ ^]^ Copyright 2019, Wiley‐VCH. b) Schematic diagram on phase transition of Ga‐coated PU. Reproduced with permission.^[^ ^44^ ^]^ Copyright 2021, Wiley‐VCH. c) Schematics and microscopic images of variable‐stiffness silicone composites containing spherical or rod‐shaped FM particles with changeable electrical and mechanical properties. Reproduced with permission.^[^ ^45^ ^]^ Copyright 2021, Wiley‐VCH. d) microhardness values with or without solid Ga as nucleating agent (Ga–Ga, pure Ga, respectively). e) Tensile stress–strain curves of Ga cured at room temperature, 4 and −20 °C; Reproduced with permission.^[^ ^47^ ^]^ Copyright 2022, Wiley‐VCH.

Buckner et al.^[^ ^43^ ^]^ proposed to use Field metal (FM) (a eutectic alloy of Bi, In, and Sn), which has a melting point of 62.5 °C, as a filler for epoxy or silicone to form a composite with enhanced variable stiffness and stretchability (**Figure** **4**a). The addition of FM can increase the stiffness of the composite when the environmental temperature is lower than the melting points of FM (*T*_m_) and the glass transition temperature (*T*_g_), which is reasonable and similar to other rigid inclusions, such as carbon black. The composites exhibited higher flexural modulus with an increase in FM when ambient temperature exceeded *T*_g_ but remained lower than Tm. Distinguished from the carbon black inclusion, the FM composite showed the lowest modulus, which was even lower than that of the epoxy. This demonstrated that the addition of FM increased the upper limit of stiffness of composite materials, reduced the lower limit of stiffness during melting, and widened the stiffness range, which forms a distinct comparison with the carbon black that the modulus ratio remains almost unchanged.

Phase transition materials can also be adhered to polymer surfaces to create programmable shapes based on the reversible rigidity. Ga particles were coated onto the 3D elastic polyurethane (PU) with the help of microwave plasma and Ga‐SH (sulfhydryl) metal bonding (Figure 4b).^[^ ^44^ ^]^ Under external temperature stimuli, the melting of the phase transition layer revealed elastic properties and allowed dramatic shape recovery. Upon cooling, the composites returned to their original shape, demonstrating shape memory.

When heated to the melting point of Ga, the shape would be recovered allowing repetitive shape recovery. By changing the weight ratio of Ga particles to PU, the modulus of the composite material increased significantly after solidification. From the stress–strain curve, when compressed to 80%, the modulus of the composite increased from 35 to 2008.9 kPa due to the strong pressure resistance of solid Ga, then it could return to 35 kPa after increasing the temperature.

In a hybrid composite system of LM and elastomers, phase transition fillers will dominate the overall mechanical property. The shape and aspect ratio of LMs also affects the conductivity, stiffness, and stiffness variation ratio of phase transition composites (Figure 4c).^[^ ^45^ ^]^ It is difficult to utilize exclusively LM particles to achieve percolation threshold of electroconductivity. The results showed that addition of particles with high aspect ratio can decrease the percolation threshold, which is due to the increased possibility of particle line contact rather than point contact and the formation of continuous electric connection. Increasing the aspect ratio of the phase change filler increased the elastic modulus in the rigid state while maintaining a low modulus in the soft state, which improved the cold‐hot modulus ratio of the composite. The authors applied strain to activate the composites by forming a longer microstructure. The results showed that an activation strain of 150% in a 55% volume ratio composite significantly increased cold state modulus and increased the cold‐hot modulus ratio by 80%.

Furthermore, in addition to particle size, the effect of oxidized shells on the surface of phase transition particles on the stiffness of the composite was investigated.^[^ ^46^ ^]^ The oxide layer of the FM had a high modulus of ≈116 GPa with an average diameter of ≈4 nm ranging from 0.003 to 16.8 nm, which was consistent with In_2_O_3_, SnO_2_, and Bi_2_O_3_. The In oxide was 90% of the total oxides and the amount of Bi oxide was negligible. After reaching the melting point of FM, the oxides with a much higher melting of ≈1910 °C could not melt, leading to an increase in the overall modulus. Experimental results showed that a larger particle size, normally with a lower content of oxides, could result in a larger modulus range. As particle size decreased, the solid oxide film formed on the particle surface further hindered the change of stiffness and increased the lower limit of stiffness. However, larger particles did not show any noticeable effectiveness in increasing the stiffness variation ratio, but produced more voids and increased the difficulty of material manufacture. Therefore, it was concluded that the maximum variable stiffness range of FM composites could be achieved with an average particle size of ≈50 µm.

The mechanical strength of LM phase transition materials depends on the solidification process. Recent research explored and compared the microhardness of solid Ga induced by two different processes: with or without a nucleating agent (Figure 4d–e).^[^ ^47^ ^]^ It is well known that the use of Ga nucleating agents allows rapid solidification of supercooled Ga at room temperature. However, the results showed that it is the nucleating agent and not the lower stimulation temperature that causes the loss of mechanical properties of solid Ga. Ga solidified with a nucleating agent was more likely to form weld marks as evidenced by the microstructure of solid Ga, which leads to a decrease in the elastic modulus and tensile strength. Therefore, nucleating agents should be used sparingly if there is a high demand for the mechanical properties of the material.

### 3.3 Phase Separation

During the phase transition process, elements form nuclei and grow until final solidification, which was largely related to the molecular dynamic and nucleation theories. The Ga‐based alloy system provides a convenient platform for observing the phase transition process. Some intriguing phase separation behaviors of the LM alloy were revealed, including surface phase separation, phase separation in the nanosystem, and unusual phase separation triggered by non‐temperature factors, such as sonication.

**Figure 5:**
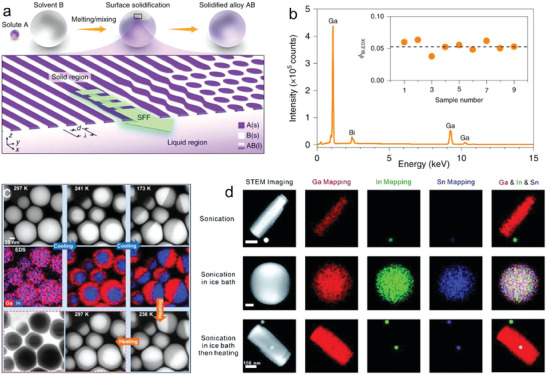
Phase separation behavior during solidification. a) Schematic illustration of phase separation and the formation of ordered patterns during phase transition of a binary alloy. b) Representative EDX spectrum of eutectic Bi–Ga (EBiGa) surface patterns. Surface Bi ratio φBi, EDX from quantitative EDX analysis. Reproduced with permission.^[^ ^38^ ^]^ Copyright 2021, Springer Nature. c) Phase separation behavior during cooling and redissolution behavior during heating of Ga‐based LM nanoparticles. Reproduced with permission.^[^ ^48^ ^]^ Copyright 2019, Elsevier. d) STEM‐EDS (Scanning transmission electron microscopy‐energy dispersive spectrometer) mapping of rods and nanospheres obtained via ultrasonic treatment of a Ga–In–Sn alloy (Galinstan) in an aqueous solution under different conditions. The scales are 100, 20, and 100 nm from top to bottom. Reproduced with permission.^[^ ^49^ ^]^ Copyright 2019, Elsevier.

Recent research by Tang et al.^[^ ^38^ ^]^ reported highly ordered phase separation on the Bi‐Ga alloy surface with different nanopatterns (**Figure** **5**a,b). In contrast to the well‐studied phase separation occurring in the inner layers of the materials, the Bi‐rich atomic layers were preferentially formed on the surface of the Bi–Ga droplets, and four unique nanopatterns were identified, namely the separate, combined, or disordered forms of the lamellar or rod‐like solidification modes. Energy dispersive X‐ray (EDX) surface analysis confirmed Bi separation on the surfaces. In addition, the authors investigated the effect of solidification conditions on phase separation. The results showed surface Bi enrichment and nanopatterns are sensitive to oxygen concentration and the Ga oxides play a significant role in surface solidification. At low oxygen concentration, the Bi separation tends to appear as a planar sheet or lateral structure different from the ordered structures. The wrinkled oxide layer can lead to the growth of disordered Bi particles along the direction of the wrinkle ridges. Thermodynamic parameters, such as the cooling rate also implement the synergetic effect of phase separation temperature and the resulting geometry.

Thus far, phase separation in nanoparticles has seldom been investigated. Tang et al.^[^ ^48^ ^]^ recently reported the phase separation behavior of Ga‐based LM alloy nanoparticles under cooling (Figure 5c). When the temperature was reduced to 206 K, phase separation was observed via EDS elemental mapping, which was manifested by the formation of a nanostructure with a solid In core and liquid Ga shell with the In core being free to move within the nanoparticle. The low solubility of In in Ga at a low temperature (<3.5%) led to the precipitation of In in the nanoparticles and the size of the In core was dependent on In concentration. When the temperature was decreased to 173 K, In was pushed to the edge and Ga solidified. When the temperature was increased to 236 K, the Ga and In phases were rearranged and Janus particles were formed. When the temperature was further increased to 293 K, complete dissolution of the In phase into the Ga phase was observed. The authors also verified that In content affected the Gibbs free energy of the nanoparticles. With sufficient In content, the resulting core‐shell structure, which consisted of Ga shell and In core can be thermodynamically stable at room temperature. In addition to binary alloy systems, ternary systems, such as GaInSn nanoparticles, were also discovered with the phase separation where bicrystal In–Sn alloy cores were observed within a Ga shell.

In contrast to the normal low‐temperature condition of material phase transition from liquid to solid state, elevated temperature have been reported to induce the formation of rigid In particles from LM nanoparticles. Lin et al.^[^ ^49^ ^]^ reported that heating triggered the transformation of LM nanoparticles into solid gallium nanorods of oxide monohydroxide (GaOOH) and the formation of In nanoparticles (Figure 5d). Usually, a high temperature is required to obtain In nanoparticles. The selective removal of Ga in the LM nanosystem enables the separation of In and dealloying. In addition, simple dealloying can also be used for the phase separation of ternary systems. After mild heating, EDS mapping verified that nanoparticles rich in Sn‐In elements were produced.

### 3.4 Tunable Adhesion

LMs exhibit tunable adhesion during phase changes and the phase state of LMs dominates their adhesion property.^[^ ^50^ ^]^ Briefly, LMs exhibit high adhesion in the solid state, while liquefied metal presents a low adhesion state, which allows their use in various applications, such as robotic grippers, transfer printing, and automatic packaging.

**Figure 6:**
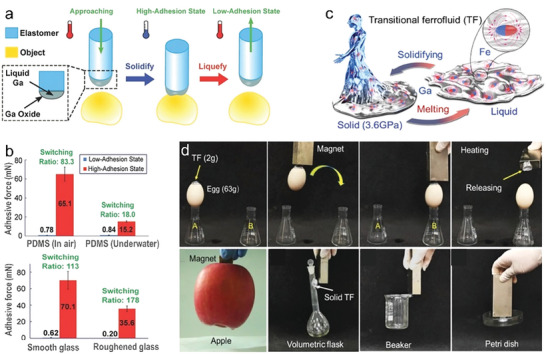
Tunable adhesion during phase transition. a) Schematic diagram of reversibly adhering an elastomer post coated with a LM layer to another object surface. b) Adhesion rates and switching ratios of objects coated with liquid Ga to different flat substrate materials on smooth polydimethylsiloxane (PDMS) surfaces under dry and wet conditions (upper) or on smooth and roughened glass surfaces under dry conditions (lower). Reproduced with permission.^[^ ^51^ ^]^ Copyright 2016, Wiley‐VCH. c) Schematic illustration of a reversible phase transition between solid and liquid in a transitional ferrofluid. d) Magnetic manipulation of non‐magnetic objects. An egg embedded with a transitional ferrofluid was controlled using magnets and the molten transitional ferrofluid was released from the egg via heating (the upper row). Magnetic manipulation performed on non‐magnetic objects of different shapes (the lower row). Reproduced with permission.^[^ ^53^ ^]^ Copyright 2021, Wiley‐VCH.

Ye et al.^[^ ^51^ ^]^ coated an elastomer with Ga and achieved the adhesion and separation of another object by tuning for maximum or minimum Ga adhesion (**Figure** **6**a,b). It is worth noting that the temperature range to distinguish between the two separate adhesive states was within 10 °C near room temperature, which was relatively easy to realize. The greatest challenge that the adhesives faced is poor adhesion under suboptimal conditions that many synthetic adhesives would exhibit impaired performances or small switching ratio in wet or rough situations due to the susceptibility to van der Waals forces. The novel adhesive method based on Ga showed strong adhesive forces (15.2–106 mN) and a high switching ratio (18–178) regardless of wet or dry conditions.

Ga, in its liquid state, can freely deform or detach from the substrate because the stress is concentrated at the edges, while Ga in the solid state will maintain a more uniform load distribution on the contact surface, generating strong adhesive forces. Despite the impressive adhesive performances, Ga still showed reduced adhesion on rough surfaces or under water. The reason for this is due to the presence of oxides. In the case of rough surfaces, the hard layer on the Ga surface limits the possibility of contact with uneven surfaces, which directly leads to a decrease in adhesion force. In underwater conditions, water can change the oxide composition, affect the interfaces, and lead to changes in the elastic modulus and yield strength, which prevents smooth contact at Ga‐substrate interfaces.

In addition, adhesion residue should be avoided during operation. The results showed that loads in excess of 3.7 kPa will result in transfer residue. The Ga oxide layer will degrade and the cohesion will fail maintain the integrity of the LM, which promotes transfer and loss of materials on the treated surfaces. By taking advantage of reversible adhesion and maneuverability, LMs have exhibited potential application in microelectromechanical systems and the manipulation technique. A demonstration of a manipulator with an adhered LM was shown in an SEM chamber where an adapted manipulator could be used to successfully lift, more and lower an object with the longest length of ≈30 µm onto a substrate, confirming the ability to manipulate objects in the the micrometer range.^[^ ^52^ ^]^

Manipulation of micron‐scale objects places increased demands on the accuracy of the system. Although preliminary experiments have confirmed the feasibility of micromanipulation, it is necessary to systematically evaluate and resolve issues related to the methods of precise temperature control, the influence of thermal deformations, and the mechanical stress‐induced charges of the electron beam.

Magnetic fields that provide precision, remoteness, and real‐time manipulation capabilities are of broad interest in magnetic sorting, drug delivery, and tumor theranostic. The combination of magnetic field control and reversible LM‐based adhesion technologies will further enable magnetic manipulation of non‐magnetic objects as well as rapid switching of the adhesive state. Wang et al.^[^ ^53^ ^]^ developed a transitional ferrofluid by incorporating Fe particles into Ga, which enabled the development of a magnetically responsive LM, since the LM itself cannot be driven by a magnetic field (Figure 6c,d). With its phase transition, the ferrofluid can be firmly adhered to substances of different shapes and be controlled by magnets. The results showed that just 10 g of transitional ferrofluid can generate 1168 N of interlocking force on an object. The Ga‐based ferrofluid also had the advantages of amorphous transformation and rearrangement to fit and manipulate various surfaces and objects compared to the traditional ferrofluid. By utilizing its magnetothermal properties, the temperature of the ferrofluid can be rapidly switched beyond its melting point, resulting in a fast transition of the adhesion state to release the object. The addition of magnetic particles improved the melting and solidification process compared to pure LM. The solidification and melting (alternating magnetic field (AMF) remote heating) rates of the Ga‐based ferrofluid were 38 and 7.6 times higher than that of pure Ga, respectively. This versatile manipulation approach can be used to operate delicate and fragile objects, such as eggs and fruits. Since the developed ferrofluid has high magnetic manipulability, the liquid residue was almost negligible.

### 3.5 Electrical Conductivity Changes

A phase change resulting in a change in shape or volume can lead to the formation of LM droplets, composites, or circuits with a change in electrical conductivity. Generally, the electrical conductivity of Ga decreases continuously with increasing temperature.^[^ ^54^ ^]^ The electrical conductivity of LM droplets can change with temperature during phase transition, in which the decomposition of LM oxides plays an important role. Thus, significant changes in electrical conductivity can be achieved in the LM droplet circuit.^[^ ^55^ ^]^ The electrical conductivity of elastomers is rather difficult to achieve. However, the addition of tunable LM additives makes it possible to control the electrical properties of composite elastic materials. The states of LMs in the elastomer will affect both mechanical properties and electrical properties. The polyurethane‐based sponge had excellent elastic recovery and durability. LM microdroplets were encapsulated to form an elastic composite.^[^ ^56^ ^]^ The resistivity of the conductive sponge doped with solid and liquid Ga exhibited excellent electrical properties under pressure on the polyurethane‐based sponge. At room temperature, the encapsulation of Ga was solid and the resistivity of the sponge showed a linear and logarithmic pressure dependence at lower and higher force, respectively. On the contrary, in the case of liquid Ga filler, a nonlinear dependence was observed due to the deformation of LM droplets. Thus, at higher pressure, the shape as well as the change in resistivity of the liquid Ga‐doped sponge was large, with the resistivity being at least one order of magnitude higher than the sponge with solid Ga. For the sample with a volume ratio of 18%, the resistivities of the solid and liquid phases reached 3.8 and 386.8 Ω m at 39.0 kPa, respectively. The phase transition of Ga from solid to liquid allows flexibility and fluidity, enabling self‐healing property and large electrical conductivity change. Stretching the solidified wire would cause the Ga filler to fracture, resulting in loss of electrical conductivity. By touching the wire with hands, the Ga fragments can be melted and healed to restore electrical conductivity, contributing to the development of stretchable electronics and soft robots.^[^ ^57^ ^]^

**Figure 7:**
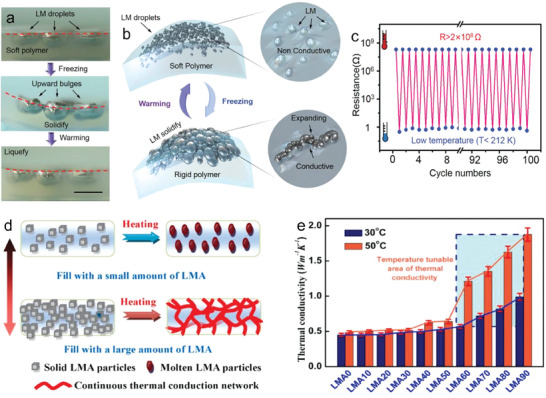
Electrical and thermal changes during phase change. a) Phase transition and volume change of LM droplet in response to temperature change (scale is 2 mm). b) The conversion mechanism of LM polymer composites between insulation and conduction. c) 100‐cycle resistance change of the LM polymer composites between electrical insulation and conduction through temperature regulation. Reproduced with permission.^[^ ^58^ ^]^ Copyright 2019, Wiley‐VCH. d) Schematic diagram of thermal conductivity change mechanism of low‐melting alloy/paraffin/olefin block copolymer thermal interface materials. e) Thermal conductivity change curves at different mass fractions and temperatures. Reproduced with permission.^[^ ^59^ ^]^ Copyright 2022, Elsevier.

Unlike most conventional materials, Ga has an abnormal volume expansion effect, similar to that of ice, in that its volume increases during solidification. This fascinating property leads to several interesting phenomena and applications in flexible electronics and soft sensors. Limited by the spontaneous formation of a Ga oxide layer, LM droplets or LM composites with droplets encapsulated by hydrogels or elastomers usually show insulation instead of conductivity. Wang et al.^[^ ^58^ ^]^ discovered that LM droplets in the frozen state can expand their shape and connect to each other inside the silicone elastomer, and developed a novel reversible transient insulator and conductor (**Figure** **7**a–c). At low temperatures, the silicone polymer became thin and rigid, allowing LM droplets to protrude, connect to each other, and form conductive paths. When the temperature was restored to room temperature, the rigid silica gel became soft again and the LM droplets melted, shrank, and re‐dispersed in the silica gel. The conductive path was broken, and the insulating state was restored. The conductivity of the polymer composite increased >10^9^ times. Similar to the above scheme principle, liquid circuits consisting of LM droplets and dimethicone were produced with conductor–insulator transition property for laser‐controlled electronics.

### 3.6 Thermal Conductivity Changes

LMs have been regarded as the existing liquid materials with the highest thermal conductivity. Its thermal property attracts broad interest in thermal management, thermal interface materials, and energy fields. The thermal conductivity of Ga increases with temperature from 11 to 270 K. An obvious drop can be measured at the melting point, which is derived from the heat absorption and the structure destruction of α‐Ga crystal. Furthermore, it returns to the original level at a higher temperature.^[^ ^54^ ^]^

With the doping of copper (Cu) particles, the thermal conductivity showed obvious enhancement. While freezing the composites, the thermal conductivity of the LM paste presented an increased trend, which was due to the reduced chaotic nature of these particles.

In applications of electronic thermal management, LM phase transitions could also cause dramatic changes in the aspect of material's thermal conductivity, which is often used to improve the heat dissipation of electronic products. Through changing the temperature, the thermal conductivity and thermal contact resistance of a LM/paraffin/olefin block copolymer thermal interface material could be adjusted (Figure 7d,e).^[^ ^59^ ^]^ The thermal contact resistance of Bi‐based low melting point alloy and paraffin wax decreased significantly with the increase in temperature. In addition, the liquid phase low melting point alloy particles were connected to reconstruct an efficient thermal conductivity path and enhance the thermal conductivity of the composite. It was found that the thermal conductivity of the composite increased with the proportion of LM solid particle filler, but it was not particularly obvious, which was due to the large interface thermal resistance. When the LM filler exceeded 60% weight ratio, the LM connection in the molten state greatly improved the thermal conductivity of the composite material (up to 1.87 W m^−1^·K^−1^). When the temperature increased, the contact state between the LM particles was also improved, and the thermal contact resistance gradually decreased.

## 4 Stimulation Methods of LM Phase Transition

As phase transition of LM brings many changes, such as morphology, stiffness, adhesion, and phase separation, a variety of methods have been developed to regulate the phase transition process and achieve functionality of LM droplets or LM composites in diverse scenarios. The phase transition of LM can be realized by many methods. Direct contact with the biological body or naturally heating/cooling under the ambient environment is the simplest and easiest way to implement phase transition. Taking advantage of diverse stimulus‐responsive properties of LM, external energies or equipment that can produce electrical, magnetic fields or thermal changes can be adopted to induce a fast transition of LMs between different modes. In addition, the addition of nucleating agents can suppress the influence of supercooling and achieve fast solidification.

### 4.1 Melting the LMs by Body or Tissue Temperature

LMs have low melting points around room temperature. Appropriate change of ambient temperature can easily cause the phase transition of the LM. To date, there is some research to adjust phase transition of the LM via the changes in ambient temperature.

**Figure 8:**
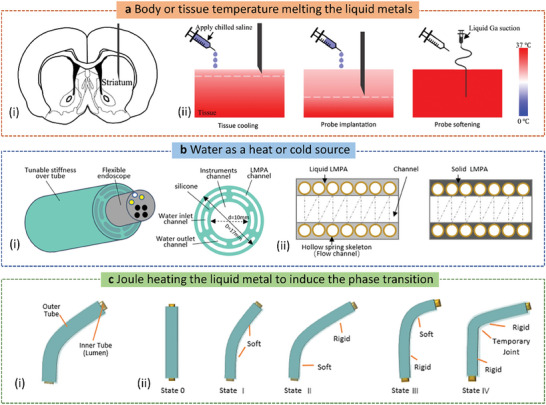
Stimulation methods of LM phase transition. a) Body or tissue temperature melting the LMs. i) Schematic diagram of the coronal brain section implanted with an adjustable stiffness probe. ii) Schematic diagram of probe implantation and soften process. Using chilled brine to cool the tissue from the surface. The white dotted line indicates a temperature of 30 °C. Reproduced with permission.^[^ ^60^ ^]^ Copyright 2019, Elsevier. b) Water as a heat or cold source. i) Structural diagram of variable stiffness tube with water inlet and outlet channel. Adapted from.^[^ ^61a^ ^]^ Copyright 2016, American Society of Mechanical Engineers. ii) Schematic diagram of the stiffness change of the spring skeleton runner: a spring‐like hollow tube was embedded in a low‐melting alloy. The flexible and rigid states of the low melting point alloy were achieved by changing the fluid in the tube. Adapted from.^[^ ^61c^ ^]^ Copyright 2021, IEEE. c) Joule heating the LM to induce the phase transition. i) Schematic diagram of the manipulator design: two flexible silicon tubes covered the inside and outside of the spring and Field's metal was poured into the space between the tubes and around the spring. ii) Five basic states of the two segmented manipulators. Adapted from.^[^ ^64^ ^]^ Copyright 2021, IEEE.

The melting point of Ga‐based materials is generally below body temperature. Therefore, the phase transition of LM can be easily achieved via directly taking advantage of the difference between room temperature and body temperature. For example, Wen et al.^[^ ^60^ ^]^ proposed a flexible neural probe made of PDMS material with a multi‐layer independent structure, wherein the third layer structure was filled with liquid Ga, which was used to realize the transition of the probe from hard to soft state at body temperature (**Figure** **8**a). The probe was able to enter the deep brain of mice in a solid state at room temperature without the help of an external carrier, and could quickly become soft inside the brain, thus reducing the damage to brain tissue. In this process, the probe could achieve a stiffness change of five orders of magnitude.

Wu et al.^[^ ^44^ ^]^ introduced a composite material with a shape memory function, which was combined with a 3D elastic polyurethane (PU) material and LM Ga coating. The temporary shape of the composite material was created by the solidification of Ga coating. When the temperature rose to near body temperature, the Ga coating melted, and its shape returned to the original state of the elastic PU material. In other words, the shape memory function of the composite material was achieved through the phase transition of the Ga coating between room temperature and body temperature.

### 4.2 Water as a Heat or Cold Source

When the melting points of the LM composites are higher than the ambient environment, the body or biological tissue is not sufficient to induce the phase transition. Additional heat or cold sources are required to provide external energy to accelerate the melting or freezing process and trigger phase state transition.^[^ ^61^ ^]^

The common methods include special structure design to allow water heating or cooling and incorporation of a heating layer or a heat dissipation layer to reduce thermal resistance and accelerate heat conduction (Figure 8b(i)).^[^ ^61a^ ^]^ According to the difference of the applied scenarios, a unique architecture was constructed to achieve the phase transformation. For example, the spring skeleton flow channel was also developed in the robotic system to achieve flexible manipulation (Figure 8b(ii)).^[^ ^61c^ ^]^ When the hot water or cold water flows over the Cu tube, the surrounding LM could be heated to a flexible state or cooled to a rigid state. The spring‐like Cu tube could not only function as a flexible skeleton of the manipulator, but also balance the weight of the manipulator. The design of spring‐like structure increased the contact area with the phase transitional metals, leading to fast mechanical transformation. In addition, attributed to the excellent thermal conductivity of the Cu, the interfacial thermal impedance could also be reduced. However, due to the intermetallic reaction between Ga and Cu, certain protection was needed to prevent corrosion. As the triggered temperature of LM phase transition would exceed the melting point and more, heat insulation protection should be considered in the body related stiffness tunable devices.

### 4.3 Joule Heating the LM to Induce the Phase Transition

Joule heating can provide external energy to heat the LM of a melting point higher than the room temperature. Normally, the Joule heating module would be put beside the phase transitional LM with intimate contact to impair the air thermal resistance.^[^ ^62^ ^]^ At the same time, the resistance should be sufficient to generate adequate heat. However, due to the high electrical conductivity of LM, direct contact with the two components would lead to a short circuit, thus, insulation wrap is required for Joule heat generation. In addition, the heating modulus could be designed as an independent layer. The nickel–chromium (Ni–Cr) alloy wire with a diameter of 0.08 mm was embedded as a heating layer.^[^ ^61b^ ^]^ After applying a current of 0.5 A for 60 s, the temperature can exceed the melting point of stiffness tunable materials, which turned soft and could be stretched by 47%. While the heating layer that is independent of the flexible layer, has limited softness and stretchability. Therefore, a soft electrical Joule heating component was proposed with a serpentine channel of liquid phase Ga–In–Sn (Galinstan) alloy. A snaking channel of liquid alloy formed a heating element and a low‐melting FM plate was embedded in an elastomer material to form a composite material with adjustable stiffness.^[^ ^62^ ^]^ A current of 6 A was applied to raise the temperature of the heater, thus melting the FM sheet to enable the composite to be easily deformed and stretched.

Despite the excellent thermal conductivity of the Cu tube, the mechanical stiffness was also increased on account of the rigid component. To achieve better thermal conduction while maintaining the softness, researchers proposed to develop a soft heat dissipation layer to reduce the interfacial thermal resistance. Many endeavors have been concentrated on the enhancement of thermal conductivity in fluid or soft materials. Compared to metal particles, such as gold (Au), silver (Ag), or Cu, Ga particles inclusion was able to regulate the mechanical mismatch between the inclusion and the surrounding polymer matrix to achieve high thermal conductivity, flexibility, and extreme toughing. Recently, Zhang et al.^[^ ^61b^ ^]^ proposed a PDMS‐based dissipation layer via mixing with EGaIn liquid drops to increase the heat dissipation rate during the cooling process. The stiffness tunable layer was made of a Ga–Fe magnetorheological fluid matrix to provide the composites with varied stiffness. Through increasing the weight ratio of EGaIn: PDMS, the cooling and heating time of the composites was shortened significantly from 44 to 28 s.

External heaters would increase the heat transfer process and response time. With excellent conductivity, direct Joule heating the phase transitional materials could bring a lot of benefits, such as a fast speed, high efficiency, and self‐sensing capability, which is critical in the design of robotic techniques and many other practical applications, such as aeronautic, civil engineering and biomedical engineering. Schubert et al.^[^ ^63^ ^]^ embedded a low melting point alloy microstructure in a soft PDMS via syringe filling to the microchannel. The melting time increased with the alloy volume. Using this model, a relative stiffness variation of more than 25 times can be achieved. It should be noted that non‐uniform volume expansion of the isotropic wires during the heating process, would lead to the resistance decrease because the cross‐sectional area normally increases faster than the length. A higher relative stiffness change with >1000 times, is able to be realized via increasing the proportion of LM. Combining with other stiffness variable materials or designing special microstructure is helpful to achieve high absolute stiffness and high strain sensitivity.

The design on a multi‐stiffness variable segment can realize continuum manipulation with safety, flexibility, and dexterity. The single segment appears as a dexterous manipulator with an open lumen for tools delivery and cooling, which is composed of two silicone flexible cannulas and a stiffness variable filler between them. The stiffness changeable part is made of spring and field's metal and was able to regulate its stiffness through Joule current flowing through the spring. Two segments connection can increase the overall diversity and degree of freedom. Though regulating the stiffness of two segments and the linking joints, five basic states of the manipulator were proposed (Figure 8c).^[^ ^64^ ^]^ The results also showed that the high power effectively reduced the transformation time, but with a lower surface temperature, which resulted from the reduced heat loss. The developed manipulator was demonstrated to withstand high payload compared to the weight. The compact design of the inner spring also enables low energy consumption and a fast transition between the soft and flexible states, which is usually for minimally invasive applications.

The integration of different stiffness variable segments can also be used in remote magnetic navigation. In order to reach the heart regions of radiofrequency ablation, a smart magnetic variable stiffness catheter based on Joule heating, including variable stiffness segments and a magnetic tip was proposed.^[^ ^65^ ^]^ Independent variable stiffness segments were able to be obtained by coiled enameled Cu wire as a heater at specific points outside the conduit. As the current flowed into the Cu wire, the LM was melted, and the catheter became soft and bent in the direction of the magnetic field to obtain the corresponding angle, resulting in complex 2D and 3D shapes.

### 4.4 Regulation of Supercooling

Due to the huge temperature difference between the melting point and the boiling point, LM could stay in liquid state for more than 2000 °C temperature range from near room temperature. Supercooling refers to the existence of a metastable liquid phase below the melting point, which is induced by the energy barriers of nucleation. Considering the subcooling effect, the liquid range can be further increased. The effect of subcooling is common in nature, in which the actual crystallization temperature is lower than the theoretical freezing point. For example, supercooled water keeps liquid state below 0 °C that brings many interesting phenomena and applications in food refrigeration, biological preservation, and chemical reactions. The subcooling of LM is obvious, which definitely affects research and application.

The thermal behavior of LM was investigated and different forms of LMs were produced under sonication, including large‐sized and small‐sized ones. In comparison with bulk LM, the particles show a different solidification performance and the subcooling effect is more significant with the size reduction.^[^ ^66^ ^]^ On the other hand, the melting properties maintain the same around the melting point. The size effect on subcooling is also demonstrated by other groups that the crystallization temperature of nano‐state EGaInSn is measured ≈−140 °C, which is much lower than that in a non‐nanostate.^[^ ^67^ ^]^ The reported lowest subcooling temperature can reach −183 °C.^[^ ^68^ ^]^ The subcooling effect of nano Ga is rather complex that liquid and solid Ga were found to coexist on a sapphire substrate at a low temperature.

The supercooling of LMs is not always a nuisance. The broadening of liquid state at a low temperature enables extreme applications in cold environments such as south or north poles, undersea, and outer space. Malakooti et al.^[^ ^69^ ^]^ developed a self‐powered bio‐electronic sleeve to monitor the heart rate under cold environments. By reducing the LM particle size and choosing suitable polymer matrix, the LM polymer composites can maintain the mechanical compliance and stretchability to −80 °C attributed to the addition of the LM fillers. This supercooling behavior was basically unaffected even when mixed with composites of different materials or with varied synthesis processes.

Influenced by supercooling, the solidification process of LM is hardly predictable. Some methods have been developed to regulate the liquid‐solid transition, including the addition of nucleating agents or interference with additional energy.

**Figure 9:**
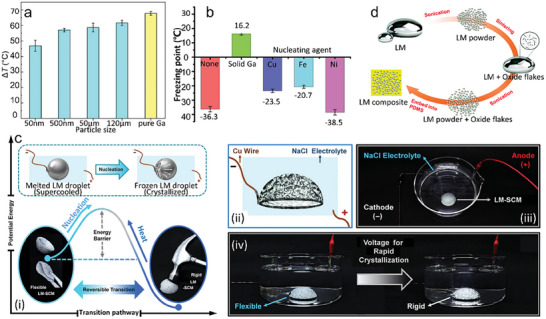
Regulation of supercooling. a) The degree of supercooling of pure Ga and its mixtures formed with Fe powder (1 wt.%) of different sizes. Reproduced with permission.^[^ ^70^ ^]^ Copyright 2019, Elsevier. b) Freezing point of liquid Ga without nucleating agents or containing different types of nucleating agents. Reproduced with permission.^[^ ^47^ ^]^ Copyright 2022, Wiley‐VCH. c) The reversible stiffness transition of the supercooled LM triggered by a small voltage (5 V) in the electrolyte solution (i). Schematic diagram (ii) and setting (iii) of rapid solidification of flexible shell LM material under voltage in salt solution. (iv) Stiffness change of shell LM material under voltage in a salt solution. Reproduced with permission.^[^ ^72^ ^]^ Copyright 2021, American Chemical Society. d) Schematic diagram of LM powder and natural oxide flakes obtained by ultrasound and sintering. Reproduced with permission.^[^ ^74^ ^]^ Copyright 2021, Royal Society of Chemistry.

As phase change materials, it is crucial to suppress the supercooling effect to minimize energy consumption. Zhang et al.^[^ ^70^ ^]^ systematically investigated the influence of various factors on LM nucleation and the supercooling effect, including thermal history, particle size, and type of nucleating agents (**Figure** **9**a). Thermal history effect refers to the influence of heating temperature on the supercooling. When the heating temperature is not high enough, the crystal embryos could be retained in the bulk LM with the aid of surface tension, further serving as nucleation sites to decrease the supercooling degree. When the potential nucleation sites are retained, the supercooling degree is influenced by the thermal history and the cooling speed. However, if the thermal history sensibility is fully eliminated, the supercooling degree is pretty stable and unaffected. The most common method of regulating the supercooling degree is introducing nucleating agents to promote the nucleation process. Small sized nucleating agents that can provide more nucleation sites are beneficial for the nucleation due to the high specific surface area, thus leading to the successful decrease of the supercooling degree. The choice of nucleating agents is critical for effective supercooling control. The amount should be suitable not too much or too little. Aggregation of the nucleating agent can impair the nucleation performance resulting from the reduction of nucleating sites and the increased proportion of heterogeneous nucleation. Nucleating agents with similar lattice constants to the liquid contribute to the nucleating process. An interesting discovery is that the wettability between the nucleating agents and the liquid would play an important role in nucleation. A better wettable behavior would lead to efficient nucleation. Among different nucleating agents, TeO_2_ was demonstrated with the strongest inhibition effect on the supercooling of Ga. Compared with pure Ga, the supercooling value decreased to 38.2 ± 5.4 °C when 0.5wt.% TeO_2_ was added as a nucleating agent.

In another study, Chakravarty et al.^[^ ^71^ ^]^ adopted an epitaxial lattice‐matching technique to evaluate the multiple cubic rock salt as nucleation catalysts for Ga and Ga‐based alloys, and demonstrated that two phases, including cubic carbide HfC and cubic nitride ZrN would realize the lowest supercooling effect that are lower than 20 and 10 °C, respectively. Furthermore, the LM with doped nucleation catalysts was verified with stability after aging for 120 days, demonstrating the practical application prospects.

Solid Ga could also be regarded as a suitable nucleating agent. Wang et al.^[^ ^47^ ^]^ showed that solid Ga as a nucleating agent could achieve the most obvious suppression of supercooling compared with metal Cu, Fe, and Ni (Figure 9b). The freezing point could be upregulated to 16.2 °C, further leading to a simple and rapid solidification of the supercooling LM. They also discovered that solid Ga as the nucleating agent would bring less energy release (≈0.014 mW mg^−1^) compared with that without a nucleating agent (1.5248 mW mg^−1^).

The application of voltage could lead to the formation of an oxide layer on the surface of the LM, resulting in a change in surface tension, which would lead to a transition in the shape of the LM.^[^ ^41^ ^]^ Combination of transformation and crystallization with solid Ga will produce an interesting phenomenon. After wetting with Ga, the anode of Cu wire acts as a nucleating agent, and at the same time the redox reaction site can trigger crystallization and irreversible deformation. The crystallization was observed from the tip of the Ga‐plated electrode after 20 s, which allowed researchers to convert liquid Ga into controllable patterns on demand.

The electrochemical reaction could also be adopted as a trigger for crystallization. When Cu electrode was connected to the supercooled Ga under applied voltage, the chemical reaction immediately occurred and CuGa_2_ formed on the surface of the Cu cathode, which could serve as a nucleating seed to trigger the crystallization of undercooled Ga (Figure 9c).^[^ ^72^ ^]^ In addition, the voltage also provided additional energy to promote the supercooling Ga to break the energy barrier, which was also beneficial for the crystallization of liquid Ga. Rapid crystallization is demonstrated at a voltage of 20 V and completed transformation into a solid state takes only 19 s. In addition, Zhang et al.^[^ ^73^ ^]^ studied the diffusion and solidification of supercooling Ga droplets during the impact process. Due to the good wettability of Cu and Ga, heterogeneous nucleation was able to be effectively induced, and the kinetic energy generated during the collision process could be converted into internal energy for solidification as the energy was required for nucleation.

Compared with Ga‐based LM, the effect of Bi‐based LM was rarely investigated. One recent study studied in detail the supercooling inhibition of Field's metal particles and composites (Figure 9d).^[^ ^74^ ^]^ Results demonstrated that various factors, such as compositions, particle size, polymer choice, and oxides would play significant roles in the supercooling effect. Similar to Ga‐based supercooling, the different scanning calorimetry (DSC) curves showed that FM with a lower size had a more significant supercooling effect. After mixing with PDMS, the size effect of FM still dominated the subcooling behavior. In addition, it was found that the natural surface oxides of BiInSnZn alloys could act as surface nucleation sites and inhibit the supercooling of the particles, while PDMS or silicone oil substrates can disrupt this effect. The supercooling of LM powder and composites could be inhibited via dispersing natural oxide sheets inside the Bi–In–Sn–Zn alloy.

### 4.5 Other Methods

In addition to the above commonly used methods for regulating the solid‐liquid transformation of LMs, other non‐contact ways, such as magnetothermal and photothermal conversion, could be considered to trigger the solid‐liquid phase transformation of LMs.

When the LM is placed in an AMF, the LM and the AMF form relative motion, which induces an electromotive force and an electric current with the same frequency but in the opposite direction inside the workpiece, which generates resistance heat when it flows through the conductor. In the study of Yang et al. ,^[^ ^75^ ^]^ AMF was used to induce heat production of non‐magnetic Ga‐In LM to achieve tumor hyperthermia. For LM particles with diameters >600 µm, the significant magnetothermal effect caused by AMF could be observed. It could be concluded that larger particle size could cause more obvious eddy heating. The heat generated by Fe_3_O_4_ and other nanoparticles under alternating magnetic fields was mainly due to Neal and Brownian relaxation. Wang et al.^[^ ^76^ ^]^ compared the temperature rise of superparamagnetic Fe oxide nanoparticles (SPION) with EGaIn at a frequency of 298 kHz and a field intensity of 120 Oe (9.6 kA m^−1^), and the results showed that the temperature increase of LM under AMF was significantly higher than that of SPION. These studies related to the magnetothermal therapy of LM suggested the large amount of heat generation and the great potential of the non‐contact method of AMF, inducing solid‐liquid phase change.

In addition, non‐contact near‐infrared laser irradiation of LM can also cause a significant increase in the temperature of LM.^[^ ^77^ ^]^ When incident light hits the materials, the vibrating electrons of the materials will convert kinetic energy into thermal energy due to the damping effect, increasing the local heat, and the temperature of metal materials will rise and spread to the surrounding areas through thermal conduction. Chechetka et al.^[^ ^78^ ^]^ prepared LM nanocapsules with an average diameter of ≈90 nm. After irradiating with a 785 nm near‐infrared laser for 5 s, the surface temperature of the LM droplet immediately rose to ≈42 °C and remained relatively stable for the next 5 min. It was observed that the heat release of LM nanocapsules in solution under laser irradiation was significantly greater than that of water. Placing 30 mg LM nanocapsules in an aqueous solution, the temperature rose by ≈30 °C after 5 min of laser irradiation, which was more significant than the effect of direct laser irradiation of LM droplets, derived from the larger light absorption surface area of the nanoscale materials. It is a feasible and convenient method to increase the LM temperature and change the LM phase via the magnetothermal or photothermal method. This avoids the additional design of complex heating devices, saving the production cost and design space.

In addition, the principle of some large refrigeration equipment such as Ar–He knife, Kangbo knife to achieve the hot and cold alternation can also be used to induce LM solid‐liquid phase change. When using an argon–helium knife to perform alternating hot and cold ablation of the tumor, argon can create a cryogenic environment, which can reduce the temperature at the probe head to −140 to −170 °C, and helium can slowly warm the temperature of the target tissue to 20–40 °C. However, unlike helium and argon, which are expensive and difficult to obtain, Kangbo Knife uses cheap nitrogen to achieve alternating hot and cold killing of unhealthy tissues, and it also extends the temperature range to 80 to −196 °C. Taking advantage of these principles to accomplish solid‐liquid phase transformation of LMs possesses great potential and broad application scenarios.

## 5 Medical Applications of Phase Transitional LMs

In the biomedical area, smart materials with stimuli‐responsive merit have gained increasing interest among drug delivery, actuators, vaccine development, and tumor treatment to actively interact with the surrounding microenvironment. In general, these regulatory methods involve a series of stimuli, such as electricity, heat, laser, sonication, and magnetic field. The requirement of biomedical devices to induce large shape or stiffness changes is growing, which is closely connected with diverse clinical demands, such as flexible mechanical joints, bone cement materials, stiffness tunable electrodes, and flexible surgical devices to conformably adapt to various cavities without damaging their mechanical strength. Phase transition LM materials can bring large modulus changes that are advantageous over other polymers, hydrogels, or shape memory alloys. Besides, the unique phase change of LM can provide other benefits in novel tumor treatment, tunable sensors, and electric skin. We have summarized the preparation, activation methods of phase transition, and the corresponding medical applications of phase transition LM in **Table** **1**.

**Table 1:** Preparation, activation, and the biomedical application of phase transition LM.

Materials	Preparation methods	Activation methods of phase transition	Biomedical applications	Ref
FM	Bi (32.5%), In (51%), Sn (16.5%) are mixed and heated to make the target alloy (FM); Flexible mechanical joint is made of the forearm, rear arm, metal chamber, thermoelectric device, and sealing ring.	Melting: thermoelectric devices heating; Solidification: nature environment cooling.	Flexible mechanical joint	[18a]
FM, silicone rubber, a spiral wire	A FM core, a tubular package made of silicone rubber and a spiral wire used as a molten alloy heater are combined to form mechanical joints.	Melting: helical conductive wire heating; Solidification: nature environment cooling.	Exoskeleton braces	[79]
Al‐NaOH‐EGaIn, BiInSn	Al‐NaOH‐EGaIn: Al powder, NaOH powder is added into EGaIn; BiInSn: Bi (32.5%), In (51%), Sn (16.5%) is mixed and heated to make the target alloy form.	Melting: adding DI water to Al‐NaOH‐EGaIn; Solidification: cooling with ice bags or cold water.	Exoskeleton braces	[18b]
Bi_35_In_48.6_Sn_16_Zn_0.4_	Bi (35%), In (48.6%), Sn (16%) and Zn (0.4%) is mixed and heated at 400 °C for 24 h to make the target alloy form.	Melting: probe heating; Solidification: nature environment cooling.	Bone repair and treatment of bone diseases	[80]
		Melting: a heating syringe; Solidification: nature environment cooling.		[18c]
Bismuth‐based metals (BBM) embolic agent	Bi_35_In_48.6_Sn_15.9_Zn_0.4_ and Ga_67_In_20.5_Sn_12.5_ at a ratio of 15:1 is mixed and heated to obtain the material.	Melting: using resistance wire wrapped around the syringe needle; Solidification: nature environment cooling.	Tumor embolization	[18d]
Ga	—	Melting: body temperature; Solidification: cryoprobe cooling or a cryostage.	Mechanical destruction of tumors	[18h]
Ga/MPs: cell membrane‐coated Ga particles (GaPs); PTX‐loaded Ga/M/PPs: Ga/MPs carrying the antitumor drug paclitaxel (PTX)	160 mg of Ga particles is mixed with 1.6 mg of C8161 cell membrane and 1.6 mg of PTX, then sonicated in the ultrasonic bath for thoroughly mixing. Then, the mixture was extruded through 5‐µm polycarbonate membrane with an avanti mini extruder to obtain the drug‐loaded and membrane‐coated particles (Ga/M/PPs).	Solidification: cryoprobe cooling.		[81]
Dynamic thermoregulating E‐skin (TE‐skin)	Galinstan: Ga (95%), In (2.5%) and Sn (2.5%) is mixed and heated to make the target alloy; Galinstan is placed in the groove of the silicone elastomer and enclosed with the extra thermally conductive silicone elastomer.	Melting: a film heater; Solidification: a thermoelectric cooler.	Thermoregulating electronic skin	[82]
An innovative multifunction fiber	EGaIn is filled into a hollow silicone rubber fiber	Melting: a heating plate; Solidification: at a low temperature environment.		[18e]
Heterogeneous substrate‐based strain sensors	PDMS layers filled with Ga in the microchannel and a graphene/carbon nanotube conductive layer are combined to obtain a strain sensor.	Melting: an inserted copper wire; Solidification: nature environment cooling.	Sensors	[83]
Ga‐microgranule‐based tunable pressure sensors (GM‐TPSs)	Ga is embedded in a microgranular configuration with uniform size in an elastomer	Melting: hot water (58 °C); Solidification: cold water (3 °C).		[18f]
Ultra‐large tunable stiffness (ULTS) probe	Platinum electrodes, GA‐filled microfluidic channels, and electrical connections are integrated onto the PDMS structure to obtain the probe.	Melting: tissue temperature heating; Solidification: freezing in vitro and chilled saline watering the tissue in vivo.	Stiffness tunable implantable electrode	[60]
LM‐based flexible electrode	Fe_3_O_4_ nano powders are doped into EGaIn.	Melting: tissue warming; Solidification: a liquid nitrogen treatment.		[ 18g]
Variable stiffness over tube based on low‐melting‐point‐alloy for endoscopic surgery	Cerrolow 117: Bi (44.7%), Pb (22.6%), In (19.1%), Sn (8.3%) and Cd (5.3%); Cerrolow 117 is embedded in three channels of the silicone rubber tube, and hot and cold water passed through the water channels of the silicone rubber tube.	Melting: hot water in the channels; Solidification: cold water in the channels.	Surgical devices (tools) with variable stiffness	[61a]
A continuous variable stiffness catheter for compliance control	Cerrolow 117: Bi (44.7%), Pb (22.6%), In (19.1%), Sn (8.3%) and Cd (5.3%); The catheter consists of a hollow working channel that is used to deliver therapeutic drugs or locate instruments at the tip of the catheter. The working channel is surrounded by a resistance coil, and the LMPA is encapsulated in an insulating layer around the resistance coil.	Melting: a conductive copper wire heating; Solidification: nature environment cooling.		[18i]

### 5.1 Flexible Mechanical Joint

Bone disease has been a major problem affecting human health for a long time. There are 206 bones in the human skeleton, each of which has a certain form and function. Bones are closely connected with each other through joints, and their form and function are mutually restricted. When the structure and function of bones and joints are damaged, it will lead to a variety of bone or joint diseases, such as osteoporosis, osteoarthritis, and stress fracture.

A joint refers to the physical connection point or fulcrum of two or more bones. The movement of the human body is mainly realized through the rotation of bones around each joint. Joint injury seriously affects the normal movement of the human body. Therefore, Deng et al.^[^ ^18a^ ^]^ constructed a flexible mechanical joint of human exoskeleton based on phase change material eutectic alloy Bi_32.5_In_51_Sn_16.5_. Flexible mechanical joints included forearms, rear arms, metal chambers, thermoelectric devices, and sealing rings. The thermoelectric device contacted the metal chamber and was responsible for heating and cooling the low melting point alloy, allowing the LM to undergo phase transitions, and the mechanical joint could easily switch between flexible and rigid states to achieve excellent mobility and load capacity. Attributed to the higher thermal conductivity, lower latent heat capacity than the commonly used paraffin wax and flexible mechanical strength, LM alloys‐based mechanical joint had advantage in the faster response speed and the higher loading capacity, which holds potential in future civilian human exoskeletons.

### 5.2 Exoskeleton Braces

**Figure 10:**
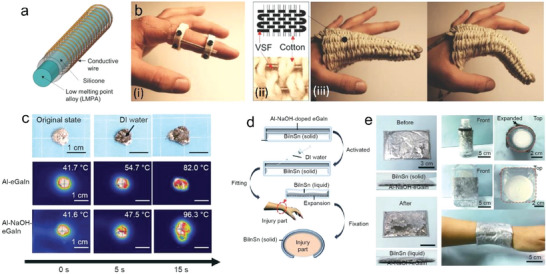
Exoskeleton braces. a) Schematic diagram of three components of a variable stiffness fiber based on a low melting point alloy: a low melting point alloy core, a silicone package, and a conductive wire. b) Finger splint prototype based on variable stiffness fiber (i) and (iii) cast prototype based on variable stiffness fiber. (ii) Fabrics consisting of variable stiffness fibers (as warp threads) and cotton fibers (as weft threads). Reproduced with permission.^[^ ^79^ ^]^ Copyright 2016, Wiley‐VCH. c) Photographs and infrared images of 1 mL of Al‐EGaIn and Al‐NaOH‐EGaIn triggered by 0.5 mL of deionized water. d) Schematic diagram of smart bandage designed by BiInSn and Al‐NaOH‐EGaIn. e) Photographs of the bandage before and after the trigger and the application on cylindrical surfaces, square surfaces, and human wrists. Reproduced with permission.^[^ ^18b^ ^]^ Copyright 2020, Elsevier.

LMs usually undergo significant stiffness changes during phase transformation and can be used in assisted bandages or exoskeleton supports by virtue of their flexibility in the liquid state and high modulus in the solid state. Tonazzini et al.^[^ ^79^ ^]^ designed a variable stiffness fiber consisting of a LM core, a silicone rubber encapsulated tube, and a heated electric conductor with molten alloy (**Figure** **10**a,b). When the LM core was heated and melted, the fiber became soft and its deformation ability achieved great improvement, facilitating the wearable rehabilitation devices. For example, a variable stiffness fiber fixed between two plastic rings could adapt to the position of the joint in its soft state and act as a structural support after curing. In addition, through weaving together with cotton thread, it is able to obtain a breathable and comfortable wearable device. The advantage of this stiffness tunable exoskeleton support is the high adaptability of different anatomical structures of humans, in which it can hold the joint in place while the bone injury occurs.

Yuan et al.^[^ ^18b^ ^]^ designed a double‐layered smart bandage filled with Al‐NaOH‐EGaIn inside and BiInSn as the supporting part on the outside (Figure 10c–e). The Al‐NaOH‐EGaIn could generate a great deal of heat when contacting DI water. The results showed that 0.5 mL water could trigger 1 ml Al‐NaOH‐EGaIn with its temperature increasing from 41.6 to 96.3 °C within 15 s. The large amount of generated heat melted the external BiInSn support layer, allowing it to fit the curves of various parts of the human surface, achieving a high degree of adaptability. A 2.5 mm double‐layer patch smart bandage system was further developed. After cooling, the external BiInSn support layer solidified, and the smart bandage could be firmly fixed to the human body or used as a temporary support in case of emergency. The internal Al‐NaOH‐EGaIn provided a soft contact surface to avoid damage to the human body. The experimental results showed that the material had an excellent adhesion effect on the surface of the human body, which was conducive to the application of smart bandages in the actual fixing process.

The tensile and bending moduli of the LM smart bandage were measured and calculated to be 11.85 MPa and 2.17 GPa, respectively, which showed much higher moduli than that of glass fiber bandages (5.24 MPa and 0.74 GPa, respectively) that were commonly used as fixed biomaterials in medical applications. LM bandages exhibited excellent reliability, rigidity, and loading capacity within 1.8 kg after solidification. When the load reached ≈2 kg, cracks began to appear in the area where the bandage met the edge of the weight due to uneven cooling. Generally, this load capacity can meet the requirements of fracture fixation in the vast majority of biomedical applications.

### 5.3 Bone Repair and Treatment of Bone Diseases

The bone defect is a common skeletal disease. Bone defect refers to a condition in which the structural integrity of the bone is destroyed due to congenital or acquired reasons. The causes of bone defects mainly include congenital factors, traumatic factors, and iatrogenic factors, such as acute bone loss, debridement after bone infection, and bone nonunion or loss of blood supply after radiotherapy or bone tumor resection. Before the advent of microsurgery and free tissue transplantation, amputation was almost the only treatment for bone defects. In recent years, with the development of medical science and technology, plenty of methods have been developed for the treatment of bone defects, including autologous bone and allograft bone transplantation, tissue engineering technology, gene therapy, auxiliary therapy of growth factor, and physical therapy. Limited to the biological tissue sources and the hospitalization cost, minimally invasive surgery has gained increased attention. Benefiting from the advancement of implanted biomaterials, the past several decades have witnessed great progress in bone defect therapy.

**Figure 11:**
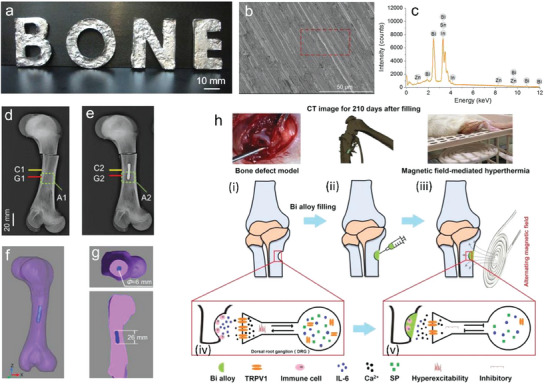
Bones repair and treatment of bone diseases. a) Formation of “BONE” shape of Bi‐based alloy cement. b) SEM image of Bi‐based alloys. c) EDS result of Bi‐based alloys. X‐ray imaging of d) partially excavated bone and e) bone filled with cement alloy. Geometric model reconstructed by f) CT sections and g) its horizontal and coronal sections. Reproduced with permission.^[^ ^80^ ^]^ Copyright 2014, Elsevier. h) Bone pain treatment. i) Bone defect model; (ii) The bone defect filled with Bi alloy using a heated syringe (green filler represents Bi alloy); (iii) The temperature of the Bi alloy was controlled remotely by an alternating magnetic field to inhibit pain; (iv) Bone defects led to increased expression of pain sensitizing factor (IL‐6, SP), pain receptor (TRPV1), and enhanced pain signaling; v) After magnetic hyperthermia, sensitizing factors and pain signals were suppressed. Reproduced with permission.^[^ ^18c^ ^]^ Copyright 2021, Wiley‐VCH.

Bone cement is a class of composite materials used to repair and strengthen bones. Previously conventional materials include polymethyl methacrylate and Ca_3_(PO_4_)_2_. However, acrylic bone cement has some limitations. Its solidification depends on chemical reactions, which usually takes a long time. There are also problems of thermal and chemical necrosis and complex revision procedures. Based on this, injectable alloy cement composed of Bi, In, Sn, and Zn was proposed with liquid‐solid phase transition capability to solve these challenging issues (**Figure** **11**a–g).^[^ ^80^ ^]^ The material had high plasticity, which is conducive to shaping. With the help of injectability and rapid solidification, alloy cement could be easily fabricated into various forms. In addition to the low melting point of 57.5 °C, its advantages also include reversible transformation, rapid solidification, low peak temperature, radiopaque, and low cytotoxicity. The solidification time of the alloy cement was related to the volume. Therefore, the alloy with a certain volume would not solidify too quickly during the operation, allowing enough operation time for the clinicians. Moreover, as the internal structural material of bone, the alloy bone cement could be used as an excellent contrast agent in radiographic imaging, which was very useful for guiding surgery and monitoring therapeutic effects.

Skeletal disorders are often accompanied by severe acute or chronic pain, which greatly impacts the physical and mental health of the patient. The combination of bone repair and hyperthermia analgesia is a good solution. He et al.^[^ ^18c^ ^]^ proposed Bi_35_In_48.6_Sn_16_Zn_0.4_ as a bone repair material, which could rapidly undergo liquid‐solid phase transformation and was conducive to in situ injection treatment of bone defects (Figure 11h). Seven months (210 days) after the bone graft, microCT imaging showed that the Bi‐based alloy did not move significantly at the bone filling site and showed good bone integration ability, thus avoiding repeated revision trauma caused by loss of bone repair materials. Importantly, the Bi alloy had strong magnetothermal responsiveness and could generate large amounts of heat in an AMF. Adjusting the power and frequency of the AMF could control the temperature of the hyperthermia. The effects of heat therapy on pain relief in mice were evaluated by paw withdrawal threshold tests and paw licking delay tests. The results showed that heat stimulation was indeed beneficial for pain relief after bone defects. Therefore, as an in situ injection material for bone repair, this Bi alloy material through wireless AMF heating is effective in chronic pain, providing a feasible and innovative solution for challenging bone diseases.

### 5.4 Tumor Embolization

Malignant tumors are a significant public health issue and have become the second most fatal disease worldwide. At present, the most common methods of cancer treatment are surgery, radiotherapy, and chemotherapy. Potential adverse effects include recurrence, severe side effects, long hospitalization time and cost, open wounds, and poor clinical effects. Therefore, the urgency for precision medicine increases gradually, which is regarded to provide minimal invasion, reduced pain, no drug resistance, and less unnecessary harm to healthy cells. Until now, emerging methods of minimally invasive tumor therapies have been explored clinically, including hyperthermia, cryoablation, and embolization therapy. The advancement of novel biomaterials and innovative technologies have greatly promoted minimally invasive tumor therapies. LMs have been explored as nanomedicine, microrobots, amorphous electrodes, and flexible patches in tumor treatment fields due to their excellent magnetothermal or photothermal conversion efficiency, biosafety, physical and chemical uniqueness, and imaging capabilities. In addition, the transition between solid and liquid states can also lead to several creative and interesting applied methods for tumor therapies.

**Figure 12:**
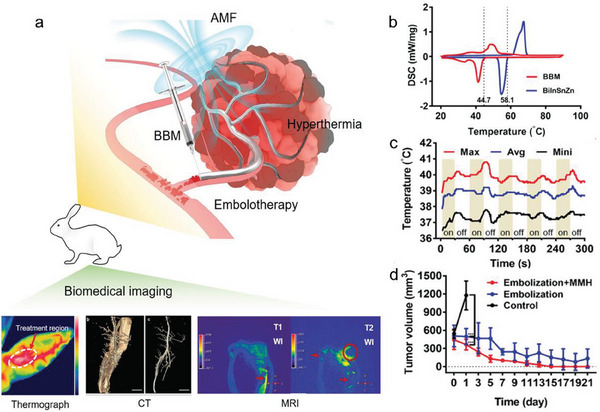
Tumor embolization treatment. a) Schematic illustration of injectable liquid embolization for tumor embolization, hyperthermia, and biomedical imaging. b) DSC curve of the metal material. c) Temperature curve of the embolized rabbit ears under magnetothermal therapy. d) Tumor growth curve of rabbit. Reproduced with permission.^[^ ^18d^ ^]^ Copyright 2022, Wiley‐VCH.

Through a starvation strategy of cutting off the nutrient source, embolization therapy introduces embolic agents into the blood supply vessels of tumor to suppress tumor growth and has been regarded as the gold treatment standard for unresectable and hypervascular liver tumors. Additionally, other clinical treatments also include arteriovenous malformation and gastrointestinal hemorrhage. At present, common embolic agents are metal spring coil, gelatin sponge, and polyvinyl alcohol. These embolic agents usually have the shortcomings of complex fabrication, insufficient embolization, or single function. Duan et al.^[^ ^18d^ ^]^ proposed a multifunctional liquid embolic agent for simultaneous embolization therapy, hyperthermia, and high‐contrast biomedical imaging (**Figure** **12**).

The phase transition temperature of the metal was ≈40 °C, allowing injection operation and reduced destruction to surrounding normal tissues. The biggest advantages are the fast embolization speed to accomplish in situ solidification and the permanent embolization capability via flowing into the micro vessels. The significant magnetothermal effect could be used for tumor hyperthermia. When the distance of the magnetic coil of the alternating magnetic field was 3 cm from the embolic agent, the temperature of the embolic agent could be controlled mainly between 40 and 50 °C, which contributes to a combined performance to tumor destruction. In addition, the embolic agent exhibited obvious signals under both computed tomography (CT) and magnetic resonance imaging (MRI), which was conducive to visualization and operation.

### 5.5 Mechanical Destruction of Tumors

**Figure 13:**
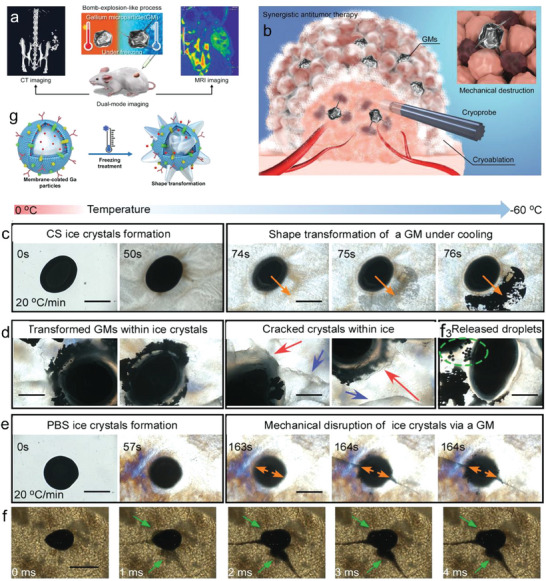
Mechanical destruction of tumors. a) Schematic diagram of Ga particles with dual‐mode imaging capability for tumor therapy. b) Ga particle enabled synergistic antitumor therapy of cryoablation and mechanical destruction. c) The formation process of chitosan ice crystals and the deformation process of Ga particles when cooled at 20 °C/ min. d) Photographs of deformation of Ga particles within chitosan ice crystals, cracked crystals within chitosan ice crystals, and droplets released around Ga microparticles (GMs). e) Photographs of Ga particles cooling in PBS solution. f) Sequential snapshots of a single Ga particle piercing into ice crystals. Reproduced with permission.^[^ ^18h^ ^]^ Copyright 2020, Wiley‐VCH. g) Schematic diagram of core‐shell structure of Ga particles wrapped in cell membrane and loaded with drugs, which changed in cactus‐like shape after freezing. Reproduced with permission.^[^ ^81^ ^]^ Copyright 2021, Elsevier.

Biological force plays a critical role in the tumor generation and evolution. However, there are limited methods on tumor mechanical destruction. In a recent study, we unconsciously discovered a unique transformation of LM microparticles under freezing and proposed a novel tumor synergistic therapy of mechanical destruction‐enhanced cryoablation (**Figure** **13**a–f).^[^ ^18h^ ^]^

Cryoablation, which continuously delivers cold energy to tumors and mainly destructs cells via intracellular and extracellular ice formation, is an effective minimally invasive treatment of tumors. Besides direct damage to cells, micro blood vessel embolization and induced anti‐tumor immune reaction can also aggravate the tissue necrosis.^[^ ^84^ ^]^ Despite the extremely low temperature in the center of the cryoprobe, incomplete ablation and tumor residue occur. The deformation of Ga particles during the drastic phase transition process can produce a powerful destruction effect, which even pierces into the solid ice crystals to achieve the mechanical destruction effect. Since LM has high thermal conductivity, dispersing Ga particles into tumor sites is beneficial for energy uniform and conformable distribution to achieve a better ablation effect. The animal results showed that synergistic anti‐tumor treatment with a better performance on tumor growth inhibition and prolonged survival rate. In addition, Ga particles were used to enhance the T2 MRI effect and mediate a dual‐mode imaging of CT and MRI, which also had great potential for the development of image diagnostics and tumor theranostics. Furthermore, Wang et al.^[^ ^81^ ^]^ decreased the particle size and coated LM particles with cell membrane (Figure 13g). When injected into the tumor site, particles were endocytosed into the endosomes of cells. The particles underwent a liquid‐solid phase transition upon cooling and exhibited invasive morphological changes, inducing the physical destruction of the endosomal membrane. Combined with the apparent X‐ray impenetrability of Ga particles, the material enabled high‐resolution in vivo imaging, which facilitated precise targeted therapy of tumors.

### 5.6 Thermoregulating Electronic Skin

The rapid development of electronic skin has promoted the fields of sensing, human‐machine interaction, flexible robotic, and healthcare systems. Inspired by the sensing function of human skin, the biomimetic electrical skin has gradually gained the capability of various mechanical perceptions, such as tactile perception, stress, and strain. However, self‐regulation of temperature is rarely explored.

**Figure 14:**
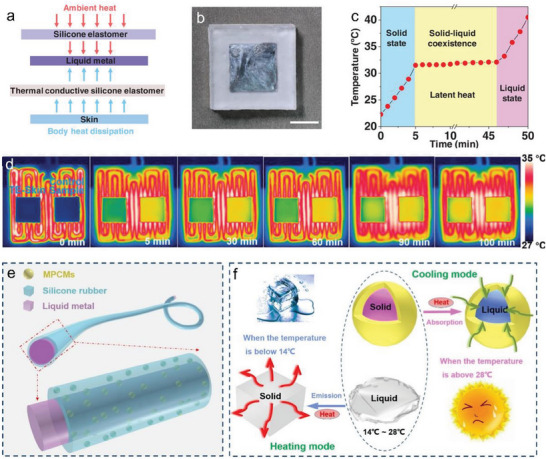
Thermoregulating electronic skin and smart fabrics. a) Schematic diagram of the temperature regulation mechanism on the dynamic electronic skin. b) Photograph of a dynamically temperature‐adjusted electronic skin. Scale bar: 1 cm. c) Temperature change of 400 g solid Galinstan in a constant 50 °C water bath. d) The thermal image of the dynamic temperature control electronic skin heated with a film heater at 35 °C. The device without the LM layer was placed on the right side of the dynamic temperature control electronic skin. Reproduced with permission.^[^ ^82^ ^]^ Copyright 2021, Wiley‐VCH. e) Schematic diagram of the multifunctional fiber structure. f) Thermal regulation mechanism diagram of the multifunctional fiber at high and low temperatures. Reproduced with permission.^[^ ^18e^ ^]^ Copyright 2021, Elsevier.

Phase change materials that can absorb or release a large amount of heat while maintaining temperature constant, have presented great application prospects in heat dissipation of large equipment, solar power storage, and energy management. With suitable phase change temperature around the human body, and favorable mechanical properties, LMs are advantageous in temperature self‐regulation in a passive mode without further energy input. A dynamic temperature‐regulated electronic skin (TE skin) with LM as a phase change material was recently developed (**Figure** **14**a–d).^[^^82]^ The LM material with a melting point of 31.2 °C was prepared by adjusting the mass ratio of Ga, In, and Sn to make its phase transition temperature within the suitable temperature range of the human body and not destroy the external protective layer of organosilicon elastomer. When the ambient temperature is higher than the comfortable temperature of human skin, heat exchange will occur between the environment and human skin. As the thermal conductivity of human skin was lower than that of the thermal conductive silicone elastomer attached to the skin surface, the heat was easily transferred from the human body to the thermal conductive silicone elastomer. Galinstan had a higher thermal conductivity and absorbed a lot of heat when melting, so the heat passing through the thermal silicon elastomer could then be absorbed by Galinstan and converted into latent heat storage. In an environmental temperature of 37 °C, the electronic skin could maintain a suitable temperature of 30.5 °C for 50 min. Due to the phase transition range and the influence of supercooling, the electronic skin temperature could be stable for 5 min under an environmental temperature of −2 °C. Overall, the electronic skin with temperature regulation capability contributes to the development of more comfortable wearable electronic devices.

Besides, smart fibers with phase change materials can also enable human temperature regulation. Ning et al.^[^ ^18e^ ^]^ prepared a new multifunctional smart fiber via filling LM electrodes in hollow silicone rubber fibers (Figure 14e,f). EGaIn with a melting point of ≈15.5 °C was selected as LM electrode and low melting point phase change material. Meanwhile, the microencapsulated phase change materials (MPCMs) with paraffin as the core and melamine resin as the shell had a higher melting point. The MPCMs were dispersed throughout the silicone fiber material, while the EGaIn was filled inside the hollow silicone fiber. The fibers obtained from this structure could achieve dual functions of heating and cooling. When the ambient temperature was below the phase transition point of EGaIn, the phase of EGaIn changed from liquid to solid and heat was released to achieve a heating mode. When the external temperature exceeded the phase transition point of the MPCMs, the phase of MPCM changed from solid to liquid, and heat was absorbed to achieve a cooling mode. Multifunctional fibers could be made into textiles, and their thermal regulation properties were compared with those of ordinary cotton fabrics. The results showed that the temperature adjustment ability of the textiles made of multifunctional fibers was better than that of ordinary cotton textiles in both high and low temperature environments.

### 5.7 Sensors

The switch on different states of phase change materials provides new ideas to promote both the sensitivity and the bandwidth in robotic skin. Electronic skin, with the capability to mimic skin perception, plays an important role in human‐machine interfaces and biomimetic areas. However, there has always been a tradeoff between sensitivity and bandwidth of pressure sensors that the highly sensitive sensors with limited bandwidth or the high‐bandwidth sensors with low sensitivity are developed. The phase change gel has been demonstrated to be used as a dielectric layer and implement high sensitivity and large bandwidth in two different modes via the switchable phase change.^[^ ^85^ ^]^ Limited by the modulus of the gels, there is still much room for improvement of the sensing capability. Since the modulus of LM can change by more than two orders of magnitude before and after phase transition, it is beneficial to realize the adjustment of the sensitivity of the strain sensor.

Mao et al.^[^ ^83^ ^]^ embedded a Ga‐filled cell into an elastomeric substrate to obtain a strain sensor that was capable of reversible mechanical modulation. The strain sensor presented the characteristics of fast response speed, high stability, and strong reconstruction ability. By adjusting the LM phase state in the cell, the response range and sensitivity of this heterogeneous substrate design would change accordingly. LM achieved higher sensitivity in the solid phase, while the liquid phase resulted in a larger response range, but with lower sensitivity. The response of the sensor to the applied strain could be dynamically and reversibly adjusted during the cycle test. Even if the tensile strain was repeatedly applied to the sensor, the amplitude of the resistance change would change according to the LM phase transition state.

**Figure 15:**
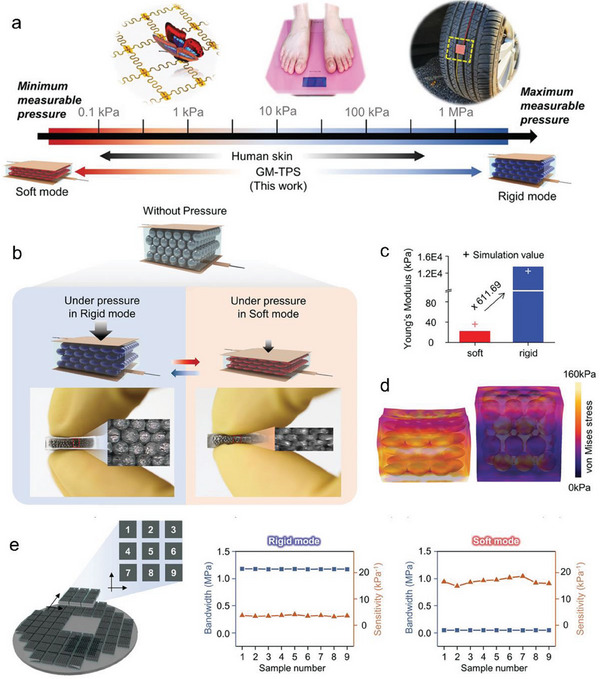
Tunable sensors. a) An adaptive robotic skin using an adjustable pressure sensor based on Ga particles and its visual sensing range. b) Schematic and optical images of an adjustable pressure sensor based on Ga particles under pressure in rigid and soft modes. c) Change of the effective elastic modulus of the sensor in rigid and soft modes. d) Von Mises stress distribution of the device in soft mode (left) and rigid mode (right) at 70 kPa simulated by finite element analysis. e) Schematic diagram of the wafer‐scale fabrication of sensor (left). Uniform bandwidth and sensitivity plots from nine sensors in rigid and soft modes (right). Reproduced with permission.^[^ ^18f^ ^]^ Copyright 2022, Wiley‐VCH.

In another work, Lee et al.^[^ ^18f^ ^]^ developed a capacitive‐type pressure sensor with Ga droplets to provide the easy conversion of the soft and rigid modes (**Figure** **15**). Benefiting from the larger modulus range of LM than that of the phase change gel, the sensor could achieve high sensitivity (16.97 kPa^−1^) in the soft state and a wide bandwidth (≈1.45 MPa) in the rigid state, enabling adaptive measurement of various pressures from 3 Pa to 1.45 MPa, which is demonstrated with enhanced pressure sensing ability beyond human skin and a broad sensing range from blood pulse to body weight.

### 5.8 Stiffness Tunable Implantable Electrode

Implantable electrodes that are inserted into deep tissue to achieve electrical or chemical signals of the biological organism have promising significance for medical diagnosis and treatment. Especially, neural electrodes and brain‐machine interface techniques have attracted broad interest among different areas and have demonstrated the therapeutic performance and motor function recovery in a variety of serious diseases, such as Parkinson's disease, epilepsy, and dyskinesia. Due to the demand of long‐term implantation, the core of the implantable electrodes lies in eliminating the mismatch between the foreign device and the surrounding soft tissue and minimizing the potential inflammatory reaction at the interface to realize precise signal manipulation and recording with reduced injury. Challenges that previously used electrodes usually suffer from, including terrible long‐term stability, poor biocompatibility and high trauma during implantation still need to be addressed.^[^ ^86^ ^]^ The use of stiffness tunable electrodes proposes a solution to the existing problem. The solid‐liquid phase change capability and good biocompatibility of LMs are beneficial to solving such problems to some extent.

**Figure 16:**
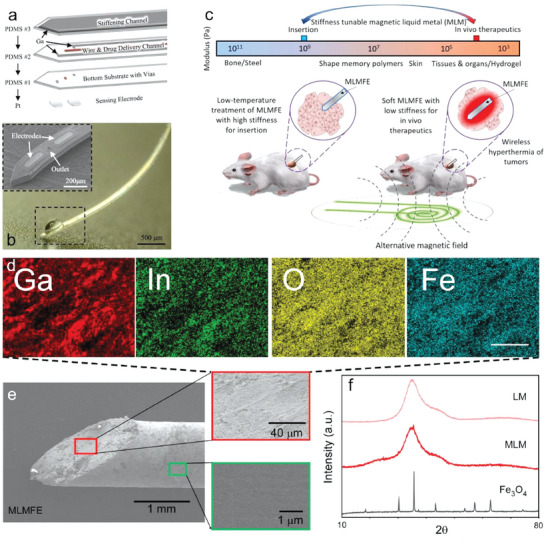
Stiffness tunable implantable electrode. a) Structural decomposition diagram of an ultra‐large tunable stiffness microprobe with 3 layers of PDMS film. b) Photographs of the ultra‐large tunable stiffness microprobe (lower). Inset: SEM images of the tip of the probe. Reproduced with permission.^[^ ^60^ ^]^ Copyright 2019, Elsevier. c) Stiffness adjustable magnetic liquid metal (MLM) and schematic diagram of the working principle. d) EDS mapping of MLM flexible electrode tip. e) SEM photos of magnetic liquid metal enabled flexible electrode (MLMFE. f) XRD of LM, MLM and Fe_3_O_4_. Reproduced with permission.^[^ ^18g^ ^]^ Copyright 2022, Elsevier.

Wen et al.^[^ ^60^ ^]^ proposed the use of a neural probe filled with liquid Ga. Due to the excellent properties of PDMS such as low Young's modulus, high tensile properties, and biocompatibility, the probe integrated platinum electrodes, microfluidic channels, and electrical connections on a 30 µm thick PDMS structure (**Figure** **16**a,b). Benefiting from the excellent solid‐liquid phase change ability of Ga at body temperature, the neural probe could achieve a change in stiffness on the order of 5 orders of magnitude. The deposition of enzymes and exclusion polymers onto a platinum electrode on the probe allowed the probe to possess the ability to perform highly sensitive electrochemical detection of neurotransmitters. Microfluidic channels then enabled the probe to deliver drugs in the vicinity of the sensing site. Thus, a variable stiffness neural probe for chemical sensing and chemical delivery was realized.

The magnetic LM obtained by doping EGaIn with Fe_3_O_4_ nanopowder had a good magnetothermal effect and could be used for magnetothermal therapy of tumors. Sun et al.^[^ ^18g^ ^]^ developed stiffness tunable electrodes of specific size and shape for flexible electrode implantation and wirelessly enhanced electromagnetic therapy (Figure 16c–f). Due to the obvious supercooling effect, the phase transition temperature of EGaIn could reach −31.60 °C. However, the phase transition temperature of the flexible electrode was significantly increased due to the doping of Fe_3_O_4_ particles, which could act as nucleating agents. Via treating it in liquid nitrogen, a strong rigid electrode was obtained for successful insertion. The range of stiffness variation of the resulting implantable electrodes could also reach 5 orders of magnitude, and the magnetothermal effect of the material was found to be enhanced with the increasing doping rate of magnetic nanoparticles. In addition, the magnetic electrode became soft after implantation, allowing wireless control of electrode position and insertion angle under the guidance of magnetic field. The results showed that this magnetic implantable electrode‐guided thermal therapy was able to provide significant tumor growth inhibition enhancement and survival time prolongation effect for long‐standing therapeutic performance.

### 5.9 Surgical Devices(Tools) with Variable Stiffness

With the continuous development and innovation of medical science and technologies, the demand for smart surgical tools is increasing in clinical practice. Surgical instruments with variable stiffness attract great interest since they can be deformed and adapted to various complex body cavities. Low melting point LMs can undergo solid‐liquid phase transitions at temperatures around human tissues, allowing for stiffness changes of these surgical devices, such as endoscopes or surgical grippers, to tackle medical challenges.

Endoscopy is an indispensable technique in minimally invasive surgery. By making a small hole in the surface of the body, the endoscope can enter deep‐sited lesion tissues or organs for observation and simple operation. It assists the surgeon to examine, diagnose, and treat the patient without creating a large wound. Endoscopic surgery has a high demand for the flexibility and stiffness of instruments. However, flexible endoscopes encounter many difficulties during the operation and the tradeoff exists between the compliant movement, including inserting and searching, and sufficient forces to exert to the target. Based on this situation, a stiffness tunable encapsulation layer is suitable to encapsulate the flexible endoscope and endow the component with flexibility and functionality. Zhao et al.^[^ ^61a^ ^]^ developed a variable stiffness tube for endoscopes, which provided different channels for multiple purposes, including LM channels and water channels. The water channel was used to circulate hot and cold water to transfer heat and change temperature, while the LM channel changed phase state under the corresponding temperature, resulting in changes in the stiffness of the whole tube. The tube produced via this method could adjust its stiffness within 20 s at a suitable temperature for human tissue. Therefore, the endoscope could not only meet the requirements of being soft enough with minimized harm to tissues and organs, but also remain firm enough to operate on the diseased site after insertion.

**Figure 17:**
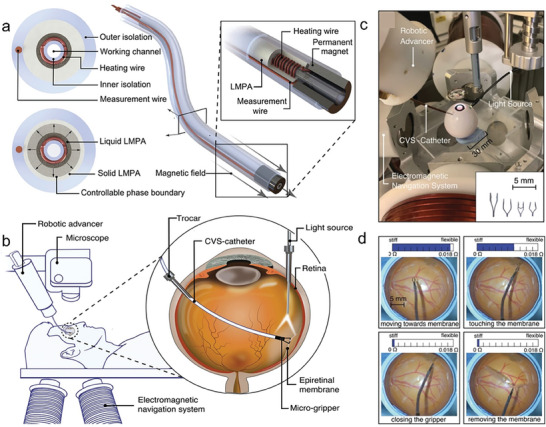
Surgical devices with variable stiffness. a) Continuous variable stiffness catheter diagram. b) Schematic diagram of the clinical setup for magnetically guided robotic membrane dissection surgery. c) Setup of magnetically guided robotic retinal detachment surgery, including an electromagnetic navigation system, robotic collinear thrusters, continuous variable stiffness catheter, a phantom eye, light source, and microscope. d) Illustration for custom micro gripper. The catheter with the micro‐clamp was moved near the prosthetic eye. The stiffness was adjusted and the clamp was closed to remove the membrane. Reproduced with permission.^[^ ^18i^ ^]^ Copyright 2021, Wiley‐VCH.

To achieve subtle and dexterous medical operations, the development of small‐scale operation tools is in high demand, however, it proposes high requirements on the materials and techniques. A recent study reported a submillimeter continuously variable stiffness catheter with high stiffness variation for compliance manipulation (**Figure** **17**a–d).^[^ ^18i^ ^]^ Through constantly adjusting the inner stiffness of the alloy and providing gradual changes in the rigidness, the catheter was able to achieve high stiffness to endow the tip with a desired force and maintain flexibility to allow safe navigation. The catheter consisted of a hollow working channel filled with LM, which could melt when heated by a resistance coil to soften the catheter. The tip of the catheter could deliver therapeutic drugs or positioning instruments. To evaluate its function, the stiffness variable tool was jointly applied with a magnetically guided tool to implement the epiretinal membrane peeling operation. The goal of the operation was to remove layers of pathological cells that had formed on top of the patient's retina due to its adverse influence on visual acuity. The tip of the catheter was equipped with a micro gripper. The catheter exhibited high flexibility in the flexible state to reach more regions of the retina. After reaching the target area, the stiffness of the catheter increased, further facilitating the manipulation of the micro‐gripper in order to pinch the cell layer to complete the surgery.

## 6 Future Outlook

Attributed to the low melting points around the human body, the phase transitional LM would bring a lot of impressive changes in shape, stiffness, adhesion, electrical or thermal conductivity. These changes of properties would further endow several extraordinary biomedical applications, including tumor mechanical destruction, embolization therapy, bone treatment, and other useful surgical devices. Due to the large supercooling of LM, the phase transition temperature can be hardly predicted. Several metals or nanomaterials have already been verified to suppress the supercooling effect. Further research efforts could be focused on the methods of precise supercooling regulation. Thus, the information, such as shape, stiffness, or adhesion, could be better controlled.

Besides the above biomedical applications, tunable adhesion or stiffness could also be used in other applications, such as transfer printing, electronic printing, or 3D metal printing.^[^ ^87^ ^]^ For example, the semi‐LM coating via welding with other fibers and solidifying could provide support for 3D structure.^[^ ^88^ ^]^ Three‐dimensional microelectrode arrays could also be quickly obtained.^[^ ^89^ ^]^ Bi‐based LM can be used as a printing material, and a syringe needle was used to pump melted LM droplets onto a flexible PDMS substrate. Using the appropriate injection speed and temperature, an average length of 270 µm needle could be easily achieved, which has the potential to be used as a skin patch. In addition, LM can also be adopted as a biocompatible electronic ink for tissue engineering.^[^ ^90^ ^]^

**Figure 18:**
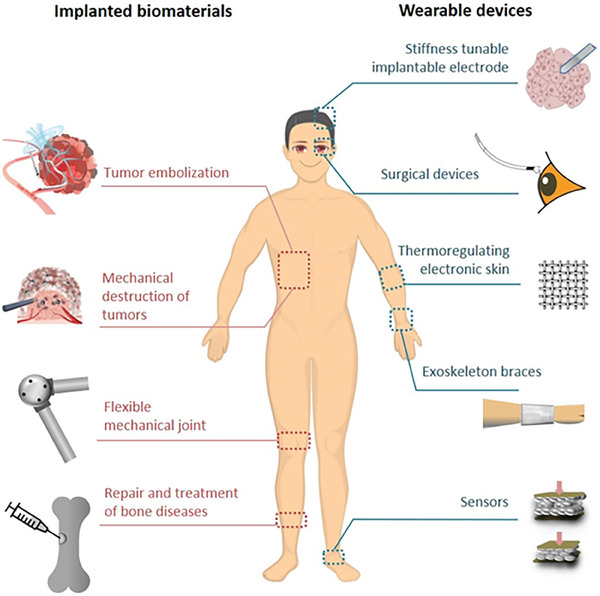
LM phase transition materials for future applications. These biomedical application scenarios are divided into two parts, including wearable devices (right) and implanted biomaterials (left).

The emerging applications of phase change LMs can cover a brunch of biomedical areas, spanning from healthcare wearables to implanted devices and biomedical micro/nano‐materials. These application scenarios are classified into two aspects (**Figure** **18**). For wearable biomedical devices, the phase transition LMs usually play a role as the stiffness tunable component in areas of flexible surgical devices, exoskeletons, electrodes, and biomedical sensors. The stiffness control and the response time depend on the regulation methods, structures, and integration besides the phase transition materials. On the other hand, the phase change LM can be used as implanted biomaterials, such as embolic agents, flexible joints, repair materials, etc.

The biosafety of LMs, whether as wearable biomedical devices or as implanted biomaterials, is of vital significance. The toxicity evaluation of LM varies with the applied modes. As wearable devices to contact with skin, such as healthcare tattoos or biomedical sensors, skin irradiation, inflammation and permeability should be seriously evaluated. If LMs are encapsulated within elastomers or by other proven biomaterials, the potential leakage and stability during usage can not be ignored. When LMs directly contact tissues or cells to exert their functionality, rigorous evaluations, including distribution, metabolization in the circulatory system, and potential damage to vital organs should be carefully studied. Moreover, in these situations, the potential heat or cold injury can not be ignored.

Custom‐designed implants could be fast fabricated via injection to further meet the clinical requirements. For LM‐based biomedical devices to succeed commercially in the healthcare sector, several problems must be solved: Compared to the existing metals or polymers, one of the biggest challenges for LMs is the high density. The development of lightweight LM is of great significance. To date, methods such as introducing low density phase or creating special porous structures have been demonstrated to effectively reduce the metal density. Further evaluations on stiffness, adhesion as well as other aspects of the lightweight LM regarding the phase transition process would be beneficial for further biomedical application scenarios; Before being put on the market, each medical equipment must pass stringent regulatory clearance procedures. Due to their special characteristics, LM‐based devices could be subject to more examination, which might lengthen the regulatory clearance process; LM‐based shape shifting devices need to be strong and dependable enough to resist regular wear and tear. Patients might suffer significant injury as a result of any flaws or failures.

**Figure 19:**
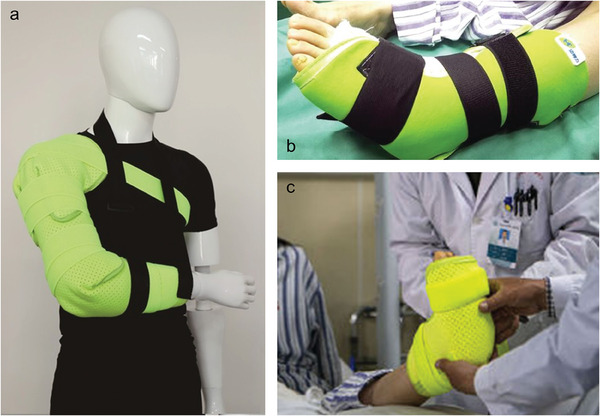
LM‐based exoskeleton fixators for clinical uses. a) LM‐based fixator for the shoulder joint. b) LM‐based fixator for the ankle and c) its clinical application for the patient with an ankle injury.

However, changes have already begun. The industrial sector is poised for a major transformation as the field of LM technology develops. In around 2013, a whole “The China Liquid Metal Valley” was formed in Yunnan Province. It is a high‐tech industrial park located in Yunnan Province, China. It is dedicated to the research, development, and production of LM‐based products, including biomedical devices, chip cooling, aerospace components, printed electronics, soft robots, and emerging functional materials. The park is equipped with state‐of‐the‐art facilities and has a strong focus on research and innovation, which aims to become a leading center for LM technology and to promote the development of the industry in China and globally. So far, a group of cutting‐edge companies have been set up. And more are on the road. Clinically, some LM‐based new conceptual biomedical equipment such as wearable health care clothes, phase transitional bone support, tumor therapy device have been developed and even translated into large‐scale clinical practices. For example, LM‐based exoskeleton fixators that can fit all the body parts, including foot, ankle, knee, waist, shoulder joint, head, and neck for various bone injury repair and bone support, have been successfully applied for patients (**Figure** **19**).

In the rapidly developing industry of LM circuitry, Liquid Wire, which was launched in 2016 in Portland, Oregon, has established itself as an industry practitioner. Utilizing a proprietary class of non‐toxic printable LMs printed on plastic and textile substrates, the company creates conformal and flexible electronic circuits. Their products are designed to enable the creation of flexible, stretchable, and conformal electronic devices that can be integrated into a wide range of applications, including wearables, medical devices, automotive electronics, and more. Liquid Wire's and other LM startup prospects in the biomedical sector are bright. Medical applications can benefit greatly from the development of flexible, elastic electronic devices that can adapt to the contour of the human body. By offering real‐time information on a patient's vital signs and other health parameters, wearable sensors and monitoring devices that may be applied directly to the skin, for instance, have the potential to transform healthcare. Additionally, implantable devices made of LM coatings might be stronger and endure longer than those made of today's materials. For patients who need implants like pacemakers, prostheses, or artificial joints, this could result in improved outcomes.

## Conflict of Interest

The authors declare no conflict of interest.
